# Genetic analysis of inflorescence and plant height components in sorghum (Panicoidae) and comparative genetics with rice (Oryzoidae)

**DOI:** 10.1186/s12870-015-0477-6

**Published:** 2015-04-19

**Authors:** Dong Zhang, Wenqian Kong, Jon Robertson, Valorie H Goff, Ethan Epps, Alexandra Kerr, Gabriel Mills, Jay Cromwell, Yelena Lugin, Christine Phillips, Andrew H Paterson

**Affiliations:** Plant Genome Mapping Laboratory, University of Georgia, Athens, GA 30602 USA; Institute of Bioinformatics, University of Georgia, Athens, GA 30602 USA; Department of Crop and Soil Sciences, University of Georgia, Athens, GA 30602 USA; Department of Plant Biology, University of Georgia, Athens, GA 30602 USA; Department of Genetics, University of Georgia, Athens, GA 30602 USA

**Keywords:** Sorghum, GWAS, Biparental QTL mapping, Inflorescence, Flowering time, Plant height, Domestication, Genetic correspondence

## Abstract

**Background:**

Domestication has played an important role in shaping characteristics of the inflorescence and plant height in cultivated cereals. Taking advantage of meta-analysis of QTLs, phylogenetic analyses in 502 diverse sorghum accessions, GWAS in a sorghum association panel (n = 354) and comparative data, we provide insight into the genetic basis of the domestication traits in sorghum and rice.

**Results:**

We performed genome-wide association studies (GWAS) on 6 traits related to inflorescence morphology and 6 traits related to plant height in sorghum, comparing the genomic regions implicated in these traits by GWAS and QTL mapping, respectively. In a search for signatures of selection, we identify genomic regions that may contribute to sorghum domestication regarding plant height, flowering time and pericarp color. Comparative studies across taxa show functionally conserved ‘hotspots’ in sorghum and rice for awn presence and pericarp color that do not appear to reflect corresponding single genes but may indicate co-regulated clusters of genes. We also reveal homoeologous regions retaining similar functions for plant height and flowering time since genome duplication an estimated 70 million years ago or more in a common ancestor of cereals. In most such homoeologous QTL pairs, only one QTL interval exhibits strong selection signals in modern sorghum.

**Conclusions:**

Intersections among QTL, GWAS and comparative data advance knowledge of genetic determinants of inflorescence and plant height components in sorghum, and add new dimensions to comparisons between sorghum and rice.

**Electronic supplementary material:**

The online version of this article (doi:10.1186/s12870-015-0477-6) contains supplementary material, which is available to authorized users.

## Background

The Sorghum genus has recently become an important botanical model for Andropogoneae grasses, by virtue of its relatively small and largely-sequenced genome, a minimum of gene duplication thanks to 70 million years of abstinence from polyploidy, and its close relationship to grasses such as maize, sugarcane and Miscanthus that have much more complex genomes [1]. Cultivated sorghum (*Sorghum bicolor*) ranks fifth in importance among the world’s grain crops, is a versatile source of food, fodder, and fuel, and possesses a great diversity of cultivated forms that may reflect its wide range of adaptation [2-4].

The ~30 year history of using linked molecular markers to dissect complex traits in plants has broadly used two complementary approaches. Conventional biparental QTL mapping [5] has been widely used and has provided foundational information that led to some successes in the identification of causal genes in many organisms. However, biparental QTL mapping generally offers relatively coarse resolution that is not sufficient to determine causative genes. Highly saturated recombination maps, multiparent advanced generation intercrosses (MAGIC) [6] or nested association mapping (NAM) [7], offer options to enhance mapping resolution of QTLs. Dramatic increases in genomic data provide rich resources with which to investigate genes and gene functions on a much finer scale than QTL mapping by taking advantage of historical accumulation of recombination events in a gene pool using ‘association genetics’ [8]. However, association mapping can require extremely high DNA marker densities to thoroughly scan a genome for genes influencing a trait, and complex measures to distinguish between artifacts such as relatedness among genotypes (especially in improved germplasm) and true evidence of functional association between a mutation and a phenotype [8-11]. Although GWAS is able to explore for causative loci on a genome-wide scale, population structure and genetic relatedness may confound associations at causative loci. Some GWAS conducted in rice and *A. thaliana* have suggested that known causative loci showed weaker signals than nearby markers [12]. In contrast, carefully designed crossing schemes in QTL mapping may be more targeted to locate relevant QTLs. Identifying intersections between results from biparental QTL mapping and association genetic data is a potentially powerful means to mitigate constraints associated with each approach, accelerating progress toward identifying specific genes that function in biological processes of relevance to agriculture.

The grass inflorescence, the primary food source for humanity, has been repeatedly selected during domestication [13]. Some well-characterized domestication traits related to the inflorescence include pericarp color of seed, seed shattering, awn length/presence and seed size/yield. Of similarly high and recurring importance in plant domestication and crop improvement are flowering time and plant height, which often show significant genetic correlation with one another [13,14].

In this study, we use GWAS to investigate 6 components of sorghum inflorescence morphology and 6 traits related to plant height, then compare GWAS-based associations to positional evidence from meta-analysis of QTL likelihood intervals. QTL meta-analysis, the comparison of multiple independent QTL studies in different germplasm and environments, is used here to provide a more comprehensive picture of the true genetic control of a trait than analysis of any single population [15], for example revealing plant height and flowering of sorghum to be genetically more complex than had been realized after more than 70 years of investigation [16]. We note that a few classically-identified loci (*dw1-dw4)* had already been compared to GWAS data [4], one of which (*dw3*) had been cloned [17], however here we address additional loci identified only by meta-analysis of QTL likelihood intervals.

Inbreeding organisms have limits to the precision in the association mapping studies [12]. Sorghum is largely inbreeding, which can result in strong LD patterns, and may lead to low genetic resolution in specific local regions along the genome. Hence, we reported the hotspots underlying 12 traits in sorghum, instead of gene candidates. The identified ‘hotspots’ provide a valuable advance toward the goal of uncovering causative variants for the trait of interest.

It is accepted widely that QTL intervals controlling common traits have non-random correspondence across and within cereals, and GWAS adds a new dimension to the ability to compare the genetic control of common traits in different cereals (and other taxa). For example, in an early comparison, the observed probabilities that seed mass (size) QTLs in sorghum, rice, and maize would correspond so frequently by chance was conservatively estimated as 0.1 to 0.8% [18]. Another early study [14] indicated that 8 of 25 regions affecting flowering of maize fall into 4 homoelogous regions. Numerous studies have shown that some orthologs across taxa have similar functions underlying common phenotypes, but other causative genes have no obvious counterparts that contribute to similar traits even in their close relatives. Thus, genetic correspondence may reflect functionally conserved ‘hotspots’ existing across taxa, but may or may not be conserved ‘genes’. Genes in a pathway exhibit significantly higher genomic clustering than expected by chance in eukaryotes [19]; for example, co-regulated clusters of genes have been implicated in QTLs affecting cotton fiber traits [20].

The common ancestor of rice, maize and sorghum experienced a whole-genome duplication (WGD; named rho) that is still readily discernible in their genomes [21], making it possible to test the hypothesis of convergent evolution across an estimated 70 million years, with the possibility of subfunctionalization among homoeologous regions. A hypothesis worthy of further exploration is that a co-regulated cluster of genes in the cereal common ancestor may have experienced gain/loss and functional divergence of some members in the subsequent 70 my of divergence, with independent domestications conferring additional functional changes in similar locations of different taxa but which are not strictly orthologous.

## Methods

### Genotype

The imputed version of ~265,000 published SNPs characterized in 971 worldwide accessions based on genotyping-by-sequencing (GBS) was employed [4]. About 72% of annotated genes contain ≥1 SNP site. A total of 228 of 354 accessions from a US sorghum association panel (SAP) [22] are converted tropical lines that are photoperiod insensitive, early maturing, and short stature phenotypes, produced via crossing exotic lines and modern U.S. cultivars. It has been demonstrated [4] that the population has sufficient power to dissect a trait, such as inflorescence architecture, that was not a target of selection in the sorghum conversion program. We used the 354 accessions from the SAP to perform GWAS. A total of 502 accessions, each characterized to a unique morphological type, were used to analyze the population structure. Wild sorghums [*Sorghum bicolor* ssp. *verticilliflorum* (L.) Moench] (n = 31) of four races, namely *aethiopicum*, *arundinaceum*, *verticilliflorum* and *virgatum*, were used to calculate expected heterozygosity for the wild population, and to compare with the heterozygosity in the 502 accessions of cultivated sorghum.

### Phenotype for GWAS

Phenotypic data for the 354 accessions in the SAP from three different growouts was utilized. We used a completely randomized design for all the 354 accessions (unreplicated trial with two observations per plot). On seed grown during 2008 in Lubbock, TX, the average RGB values for pericarp color for each genotype were determined from images of the surfaces of 5 seeds. Using the conversion formula of RGB→CIE-L*ab, RGB was transformed to CIE-L*ab color space. RGB is summarized by three values: R, G and B, and CIE-L*ab is summarized by: L, a and b. From growouts in 2009 (seeds sowed on May 19th) and 2010 (seeds sowed on May 26th), near Watkinsville GA, we took two representative samples (from each plot) per genotype in each year for the following measurements. Awn presence: presence or absence of awn, with 2 for abundant, 1 for occasional, and 0 for absent; base-flag length: measured in cm, from the plant base to the flag leaf; flag-rachis length: measured in cm, from the flag leaf to the top node, with positive values indicating the flag leaf below the rachis and negative values indicating the flag leaf above the rachis; inflorescence length: measured in cm, from the rachis to the top of inflorescence; inflorescence width: measured in cm, at the widest point; nodes: the number of nodes; whorls: the number of whorls; dry inflorescence weight; dry stalk weight; total plant height; and flowering time: the average number of days for the first five heads to flower, from the planting date. For traits measured on two samples, we used the average values per genotype to assess heritability and phenotypic correlations. Since the raw data in all the quantitative traits in our study is distributed in near-normal fashion, we conducted GWAS by using the data without transformation. Considering that data combined across years may weaken association levels for those traits with low heritability, we performed GWAS on data for each year individually. The phenotypic correlations determined by Pearson correlation are available from Additional file 1: Table S1.

### QTL mapping

QTL confidence intervals were from two resources. (1) We compiled 1-LOD likelihood intervals, which have been identified to underlie any of the 12 traits from published literature [13,14,18,23-29]. (2) We used a recombinant inbred line (RIL) from an interspecific cross between *S. bicolor* and *S. propinquum* (BTxSP), the widest cross that can be made with *S. bicolor* using conventional techniques, containing 141 loci on 10 linkage groups collectively spanning 773.1 cM to map the confidence intervals for base-flag length, flowering time, nodes and total plant height [29] for 2010 and 2011 data. Phenotypic data were collected with two replications per genotype in both 2010 and 2011. A LOD score of 2.5 was used for QTL detection. The methods for anchoring QTL intervals to the reference genome have been discussed in Zhang et al., 2013 [16]. Briefly, based on colinearity between genetic and physical positions of markers, a QTL region is delineated by two flanking markers nearest to the likelihood peak that have alignment information (BLASTN hits). Genomic positions for intervals are available from Additonal file 2: Table S2.

### GWAS

The Compressed Mixed Linear Model (CMLM) involves genetic marker-based kinship matrix modeling of random effects, used jointly with population structure estimated by principal components analysis (PCA) to model fixed effects [30-32]. Since more extensive genetic variations may confer a phylogeny closer to the true one, the total of 265,487 SNPs in the SAP were used to analyze population structure. The compression level and optimal number of principal components that adequately explain population structure were previously determined by the Genomic Association and Prediction Integrated Tool [30-32]. Log quantile–quantile (QQ) P-value plots for 265,487 single-SNP tests of association (Additional file 2: Figure S1-S3) implied that there were few systematic sources of spurious association using CMLM, noting the close adherence of P values to the null hypothesis over the most of the range. Genomic positions for identified hotspots are available from Additional file 1: Table S3.

### Significance threshold

We performed Bonferroni-like multiple testing correction [33] to determine significance thresholds for GWAS. Instead of 265,487 independent tests assumed in the Bonferroni method, the total number of tests was estimated by using the average extent of LD across the genome. On average, LD decays to background levels (*r*^2^< 0.1) within 150 kb in the current GBS data [4]. The effective number of independent tests was defined as LD bins [reference genome size (730 Mb)/average LD extent (150 kb)]. Given 0.05 as the desired experiment wide probability of type I error, a significance cutoff within about an order of magnitude of 10^-5^ was estimated.

### Overlap between QTL and heterozygosity reduction

Since the hypergeometric probability distribution (sampling without replacement) $$ p=\frac{\left(\begin{array}{l}1\\ {}m\end{array}\right)\left(\begin{array}{l}n-1\\ {}s-m\end{array}\right)}{\left(\begin{array}{l}n\\ {}s\end{array}\right)} $$ can assess the correspondence between QTLs [14,23], we used the hypergeometric probability distribution to evaluate genetic overlap between plant height/flowering time QTLs and significant heterozygosity reduction in wild sorghum. *n* is the total number of intervals (defined as 30 cM, approximating a QTL likelihood interval) along the whole genome; *l* is the number of intervals having significant heterozygosity reduction; *s* is the number of intervals having plant height/flowering time QTL; *m* is the number of intervals having both features (overlapping intervals). The purpose of this test is to show that biparental QTL mapping may capture genetic variations for domestication traits that were evolved from wild sorghum and of importance in the history of sorghum selection. Thus, we used QTL intervals for plant height/flowering time that were only determined by biparental QTL mapping. The regions with the largest 1% of heterozygosity reduction values were selected for testing. We utilized alignment between a high-density genetic recombination map [34] and the sorghum reference genome [1] to unify the genetic positions of QTLs and heterozygosity reduction regions (based on windows of 500 consecutive SNPs).

### Reference genomes

The gene annotations refer to JGI annotation release Sbi1.4 [1] and Michigan State University Rice Genome Annotation Project (MSU-RGAP release 7) [35].

## Results and discussion

### Phylogenetic relationships of five main sorghum races

Morris et al used a genome-wide SNP map to explore the population structure of 971 sorghum accessions, and illustrated the differentiation of their geographic origins [4]. However, several intriguing questions remain, for example: (1) what is the most primitive sorghum type?, and (2) how many independent domestications has sorghum experienced?

**Figure 1:**
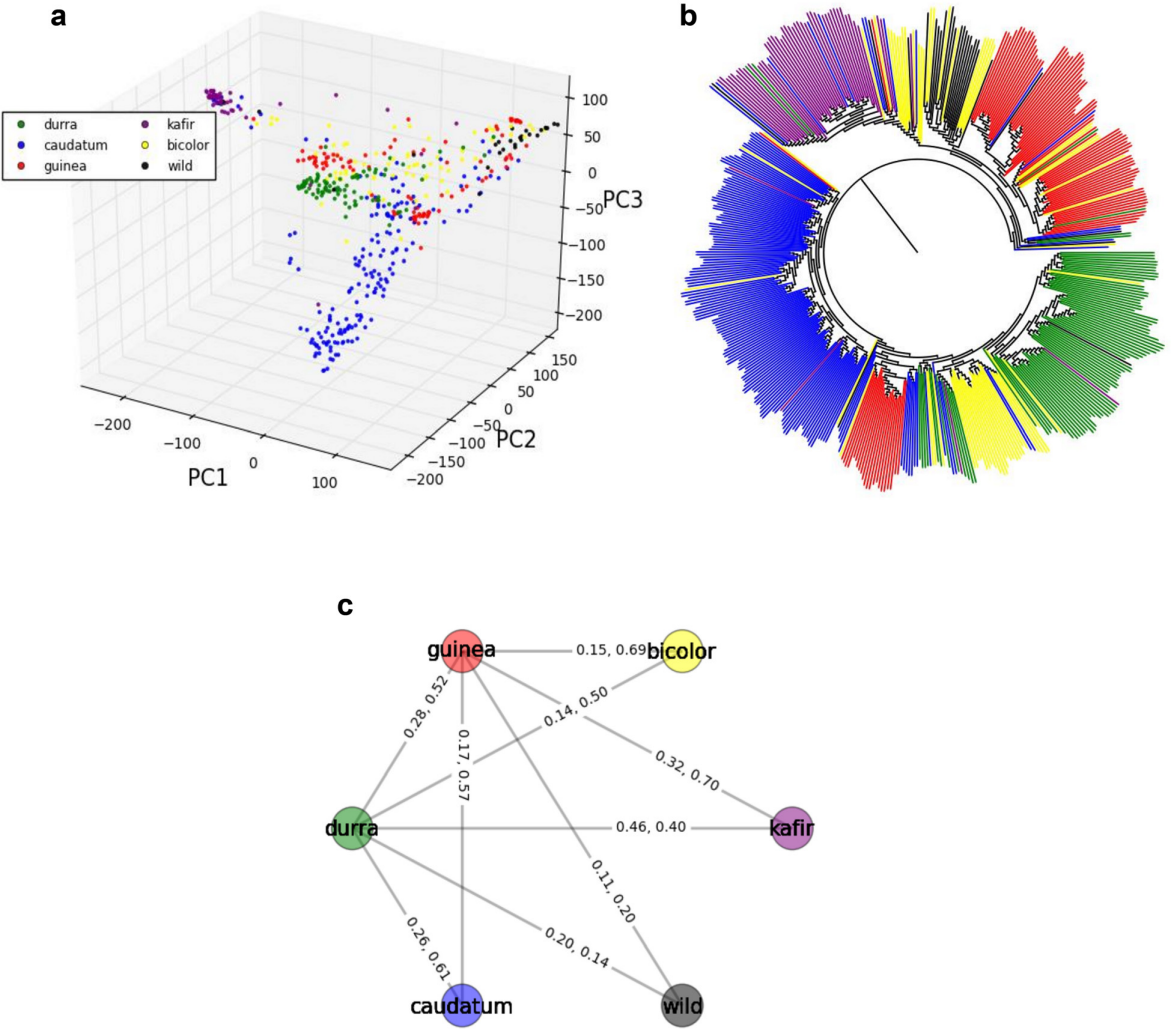
Population structure of 502 worldwide sorghum accessions. 476 belong to the five main cultivated races and 26 are wild types. **(a)** PCA plots of the first three components for 265,487 SNPs. The five main cultivated races and the wild type are color-coded. **(b)** Neighbor-joining tree of 502 sorghum accessions. **(c)** Population differentiations and frequencies of common two-locus haplotypes for 100 SNPs adjacent to the *Sh1* gene [2] for pairs of populations. All the connections for guinea and durra are shown. The F_ST_ values paired with frequencies of common two-locus haplotypes are indicated.

It is generally accepted that the domestication of sorghum started in Africa. Bicolor, guinea, caudatum, durra and kafir are five main morphological types that are well recognized to represent genetic diversity in the cultivated sorghum. Wild sorghum [*Sorghum bicolor* ssp. *verticilliflorum* (L.) Moench] including four races (namely *aethiopicum*, *arundinaceum*, *verticilliflorum* and *virgatum*), is the progenitor of the cultivated sorghum [*Sorghum bicolor* (L.) Moench] [36,37]. The phylogenetic relatedness in sorghum races has been discussed in several studies [38-41], but ambiguous clustering patterns have often been found, part of which may be attributable to the limitations of either low-density markers or small population size. To re-investigate inferences drawn before, we focused on a subset of 502 accessions, in which 471 are each characterized uniquely to one and only one of the five primary cultivated races and 31 are wild types. Both the phylogenetic tree and the PCA plots indicate that bicolor is the most primitive race, based on having close phylogenetic relationship with wild types (Figure 1a and b). The level of population differentiation, fixation index (F_ST_), was measured between wild types and each of five primary types. Race bicolor (F_ST_ = 0.04) exhibits closer genetic relationship with wild sorghums than any of the other 4 primary races (F_ST_ (guinea-wild) = 0.11, (durra-wild) = 0.20, (kafir-wild) = 0.33, (caudatum-wild) = 0.14), with guinea and caudatum apparently representing early derivatives. A large block of bicolor accessions are intermediate among durra types, potentially consistent with an ancestral relationship (Figure 1b), noting that F_ST_ (0.14) supports small population differentiation between bicolor and durra. Likewise, a large block of guinea accessions clustering within caudatum may suggest another derivation in the history of sorghum selection, also supported by evidence of minimal population differentiation (F_ST_ = 0.17). Races caudatum, durra and kafir show clustering patterns that are substantially distinct from one another, save for occasional single accessions that could be misclassifications (Figure 1b). Pairs of these 3 sorghum races show relatively high levels of population differentiation [F_ST_ (durra-caudatum) = 0.26, (durra-kafir) = 0.46, (caudatum-kafir) = 0.33]. Although the first three components of PCA are able to explain 89.1% of the total genetic variance, ambiguous clustering patterns are still occasionally observed, especially for the two primitive races bicolor and guinea. Both the PCA plot and neighbor-joining tree show quite similar clustering patterns by using the complete SNP set (265,487 SNPs) (Figure 1) and a set including more informative SNPs (missing rate ≤ 50% and MAF ≥ 0.02) (Additional file 2: Figure S4). The complex history of diffusion and selection in sorghum may confound phylogenetic inference using the clustering patterns. The phylogenetic relationships of sorghum races are still open to questions that may be more accurately addressed with growing genotype data.

Using representative SNPs across the *Shattering1* (*Sh1*) gene, a key domestication locus [2], a recent discovery revealed four haplotypes, in which three represent non-shattering forms. Specifically, guinea and durra share a common haplotype (SC265); a second haplotype (Tx623) is prevalent in kafir; and a third haplotype (Tx430) is dominant in caudatum. Three non-shattering haplotypes strongly suggest at least three domestication episodes for reduced shattering in the five main sorghum races [2,3]. In general, domestication loci can exhibit very strong LD, because of reduction in genetic divergence. Unusually high genetic divergence at *Sh1* in cultivated sorghum races may explain why the surrounding region does not show stronger LD than the background level. The clustering pattern in the neighbor-joining tree and F_ST_ values support the inference that kafir and caudatum have experienced two independent domestication events for non-shattering. However, is the non-shattering allele derived from a common ancestor of guinea and durra? Generally, we did not observe a close phylogenetic relationship between guinea and durra on the basis of the clustering patterns of the genetic tree (Figure 1b), and the large value of F_ST_ (0.28). This relatively high level of genetic differentiation implies that guinea and durra may have experienced convergent domestication events. The types of haplotypes resulting from selection could be restricted by the limited number of representative SNPs detected across *Sh1*. To overcome this limitation, we examined the region adjacent to *Sh1* to provide a pool enriched by SNPs that are tightly linked to the shattering locus. Since *Sh1* was not genotyped in the current data set, it would be challenging to know the exact number of SNP sites that are linked to *Sh1*. Instead, using 100 SNPs in a 200 kb region adjacent to *Sh1*, we examined haplotypes in consecutive pairs of loci for each sorghum population. The ratio of common haplotypes/total haplotypes was calculated for each pair of sorghum types. Among the 9 possible pairwise comparisons of populations (Figure 1c), guinea-durra only shows a modest frequency (0.52) of common two-locus haplotypes, that is lower than guinea-bicolor (0.69), guinea-caudatum (0.57), guinea-kafir (0.70) and durra-caudatum (0.61). Both guinea and durra have extremely low frequencies (0.20 and 0.14) of common haplotypes with wild sorghum. Below, using two additional domestication genes mapped herein, we further investigate the hypothesis that guinea and durra may have experienced independent domestication events but achieved non-shattering by convergence.

### Meta-analysis of sorghum QTLs

Biparental QTL mapping is based on the principle that genes and linked DNA markers largely co-segregate during meiosis save for occasional recombination events, thus allowing their analysis in the progeny. The limited number of recombination events captured in progeny of recent crosses may result in QTL likelihood intervals that contain dozens or even hundreds of genes. Further, the environment and parental lines used in a cross can limit the power to accurately estimate the number of QTLs and magnitude of their effects. Using a database that we have recently described [16], we compiled l-LOD QTL likelihood intervals resulting from 11 independent biparental QTL mapping studies to yield a more complete picture of the genetic control of a trait than could be obtained from any individual study.

**Figure 2:**
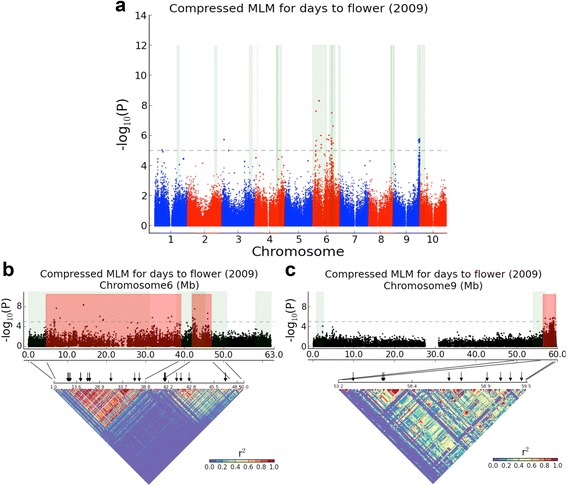
GWAS for flowering time in 2009. **(a)** Genome-wide Manhattan plot of CMLM. Significance threshold is denoted by the gray dashed line. The 10 sorghum chromosomes are plotted against the negative base-10 logarithm of the association P value. Areas highlighted in green indicate confidence intervals for flowering time determined by QTL mapping. **(b)** Chromosome Sb06 Manhattan plot of CMLM (top). Red areas show hotspots for 2009 flowering time identified by association mapping. Linkage disequilibrium (*r* ^2^ were calculated from SNPs with association *p* ≤ 0.05 and missing data ≤ 50%) matrices (bottom) are plotted for regions denoted by anchoring lines. Regions of strong LD are shown in red. Significant association markers are denoted by black arrows. **(c)** Chromosome Sb09 Manhattan plot of CMLM.

The pericentromeric region of sorghum chromosome 6 (Sb06) has repeatedly shown evidence of genetic control of plant height (Additional file 2: Figure S11), and was thought to harbor two classic dwarfing genes (*dw2* and *dw4*) [4]. The general lack of recombination in this region allowed QTL confidence intervals to cross centromeres and cover broad genomic areas. Two other regions repeatedly associated with plant height are in the euchromatin of Sb07 and Sb09 (Additional file 2: Figure S11), which were considered to contain *dw3* and *dw1* respectively [4]. An additional 9 nonoverlapping regions in the sorghum genome containing height QTLs (Additional file 2: Figure S11) show that genetic control of sorghum plant height involves substantially more than the four genes reported in classical studies [42]. Likewise, 14 flowering QTL likelihood intervals published in six studies fall into at least 11 non-overlapping regions (Figure 2 and Additional file 2: Figure S12), strongly indicating far more than the classically suggested six genes, *Maturity1* (*Ma1*) to *Ma6*, in genetic control of sorghum flowering time [16,42,43]. Flowering time and plant height show significant genetic correlations on chromosomes Sb01, Sb03, Sb04, Sb06, and Sb09, indicating that their inheritance is linked either functionally (pleiotropy) or physically (linkage disequilibrium) [13].

### GWAS for sorghum

To dissect the genetic basis of 12 traits in sorghum by GWAS, we used a compressed mixed linear model (CMLM) [30,32] to assess evidence of phenotype-genotype association. Three steps were taken into consideration: (1) determination of significance thresholds for association, (2) identification of linkage disequilibrium (LD) regions for significant association signal and (3) positive control of association.

A major issue with genome scans, which involve many thousands of independent statistical tests, is multiple testing. The Bonferroni method approximates the significance cutoff for an overall (i.e. genome-wide) 5% probability of type I error as 0.05/265,487 = 1.89 × 10^−7^ in our studies. However, this method has been criticized for its stringency [44] owing to the fact that genotype at some SNP loci are correlated thus are not independent hypotheses. Sorghum is largely inbreeding, which can result in strong LD patterns along the genome, so that an appropriate significance threshold may be larger than 1.89 × 10^−7^. Here, we used the quantified average LD information in sorghum to adjust the Bonferroni correction [33] (See details in Methods). A significance cutoff within about an order of magnitude of 10^−5^ is inferred to balance an acceptable false positive rate with sufficient power to detect true associations.

It is also important to determine LD with single SNP association, especially when causative variants are not genotyped (or at least not known). On the basis of pairwise measures of LD (*r*^2^), ‘block-like’ structures can be visually apparent. It is now well understood that the extent of LD in the pericentromeric region, which experiences relatively little recombination, is greater than in the euchromatin, which experiences more frequent recombination. A long LD block with association signals is most likely to contribute striking features to the ‘skyline’ of a genome-wide Manhattan plot.

Known genes and biparental QTL intervals that have been identified previously are useful to assess association validity. If knowledge of such candidate genes/intervals is limited in the species of interest, information from closely related species might be utilized, using synteny-based approaches to deduce orthology. In addition to the compilation of QTL likelihood intervals and *Dwarf1*(*Dw1*)-*Dw4* loci [4] in sorghum, sorghum maturity (*Ma*) genes *Ma1* [14] and *Ma6* [45], and genes *yellow seed1* (*y1*) [46,47] and *Tannin1* (*Tan1*) [48] for sorghum pericarp color provide positive controls for GWAS.

### Traits related to the sorghum inflorescence

We investigated 6 properties of the sorghum inflorescence, including awn presence, pericarp color, dry inflorescence weight, inflorescence length and width, and whorl number.

**Figure 3:**
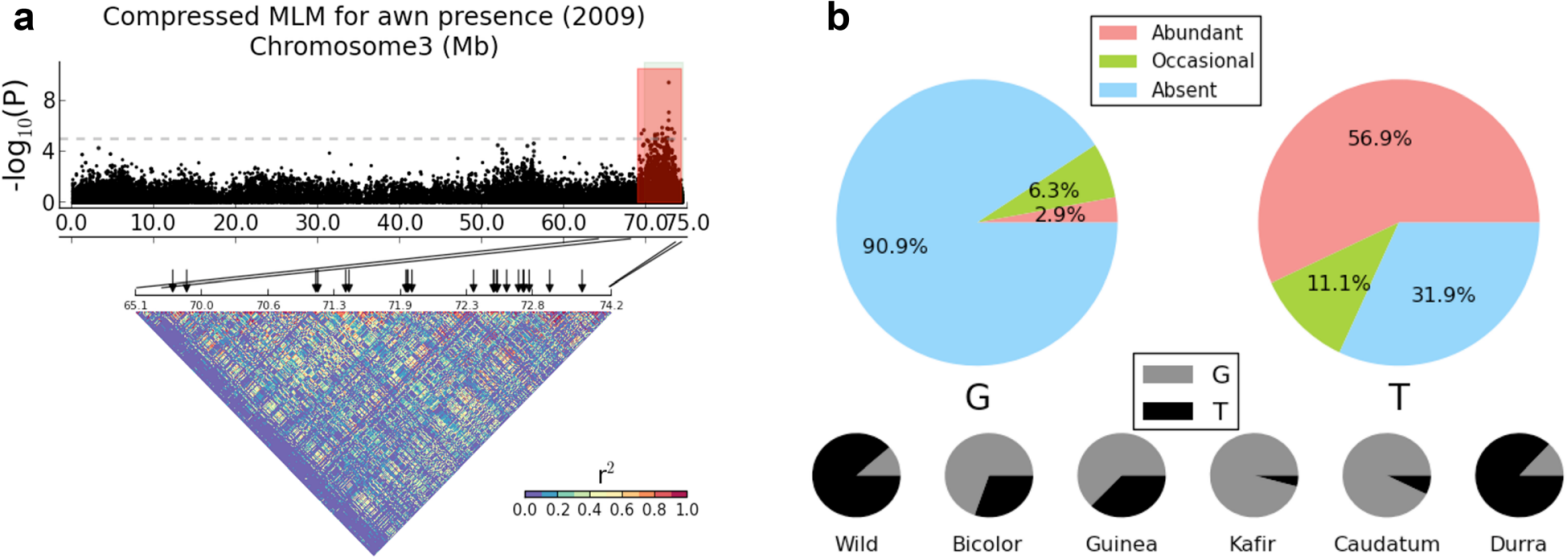
The spectrum of awn presence variation and allele frequencies at locus “S3_72702502” on chromosome Sb03. **(a)** Chromosome Sb03 Manhattan plot for 2009 awn presence in sorghum is plotted with the hotspot (red area) identified by GWAS, with the prior interval (green area) determined by QTL mapping [24], and with the LD pattern determined by *r* ^2^. Significant association markers are denoted by black arrows. **(b)** Three types of awn classification, which are “abundant”, “occasional” and “absent” are color-coded in their frequencies plots for alleles “G” and “T”. The allele frequencies are plotted for the five main cultivated races and wild sorghum.

**Figure 4:**
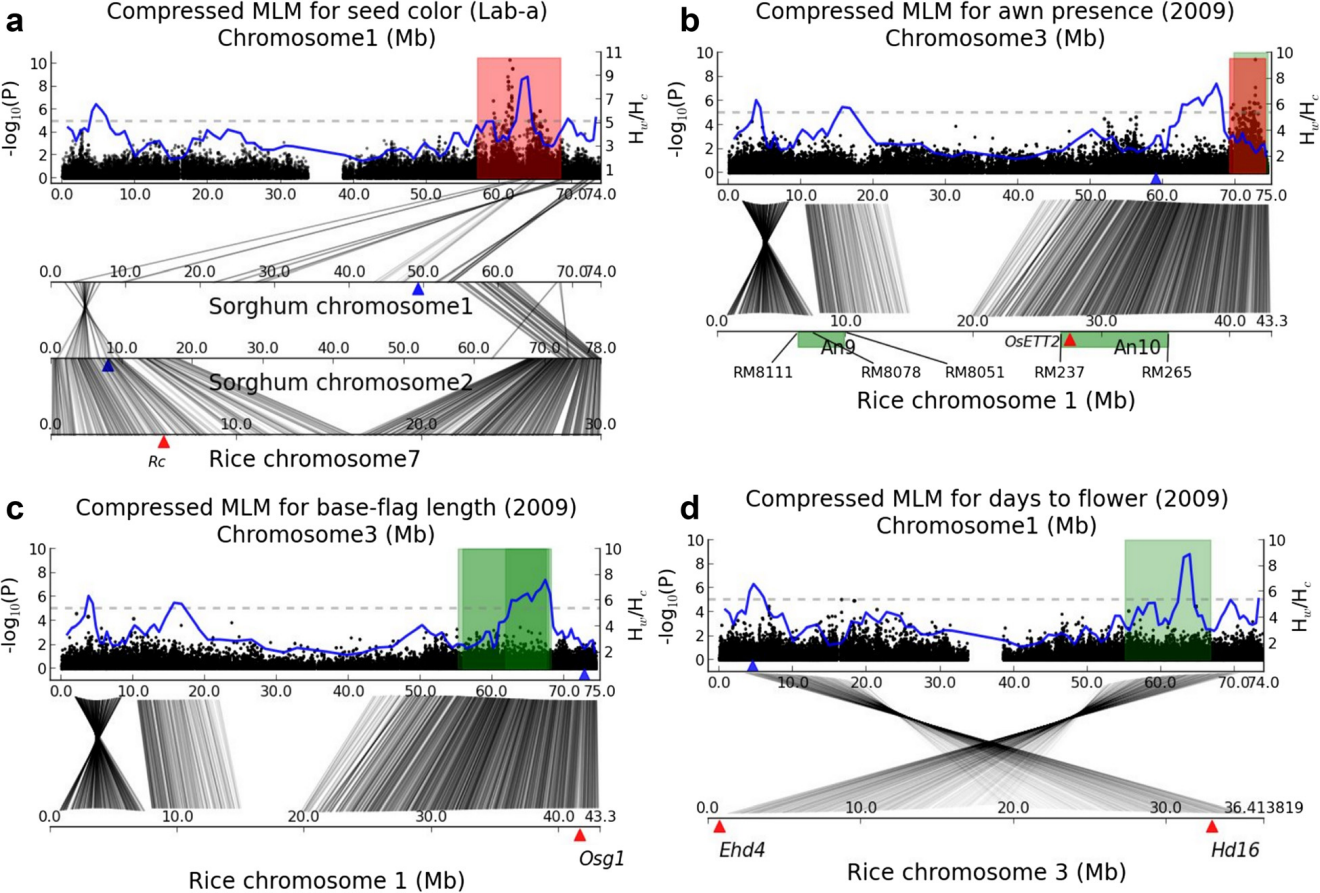
Genetic correspondence across taxa. **(a)** Chromosome Sb01 Manhattan plot for pericarp color (a value of L*ab model) of sorghum is plotted with the hotspot (red area) identified by GWAS, and with the curve of H_w_/H_c_ ratios. The *Rc* [52] gene is denoted by a red triangle. Two sorghum homologs of *Rc* on chromosomes Sb01 and Sb02 are denoted by blue triangles. Gray connecting lines indicate pairs of duplicated genes. **(b)** Chromosome Sb03 Manhattan plot for 2010 awn presence in sorghum is plotted with the hotspot (red area) identified by GWAS, and with the prior interval (green area) determined by QTL mapping [24]. Rice awns co-segregated with SSR marker RM8078 tightly linked to An9 on chromosome Os01 [63]. The interval An10 for rice awn [63] was associated with SSR markers RM265 and RM237 on chromosome Os01. The *OsETT2* [64] gene is denoted by a red triangle. Sorghum ortholog of *OsETT2* is indicated by a blue triangle. **(c)** The genomic interval (~55 Mb-67 Mb) on chromosome Sb03 is implicated by three linkage studies [14,26,28] to affect plant height and flowering time, but doesn’t harbor association signal in our GWAS. The *Osg1* [66] gene and its sorghum ortholog are indicated by a red and a blue triangle individually. **(d)** The genomic interval (~58 Mb-64 Mb) on chromosome Sb01 is implicated by two linkage studies [24,26] to control plant height, but doesn’t harbor association signal in our GWAS. The genes *Ehd4* [67] and *Hd16* [68] are indicated by red triangles, and sorghum ortholog of *Hd16* is denoted by a blue triangle.

Using a *S. bicolor* intraspecific map (BTxIS), Hart et al., [24] identified an interval controlling the presence of awns in euchromatin near the 3′ end of chromosome Sb03. The most striking association based on GWAS of awn presence for two years was in the genetically mapped interval (Figure 3a, Additional file 2: Figure S5 and Figure 4b). Both mapping strategies achieve similar genetic resolution, with intervals spanning ~4.7 megabases (Mb). We found 10 additional significant association hotspots in 2009 and 7 in 2010, none of which are consistent in the two years, which could represent modifiers affected by environment, or false positive associations.

Inheritance studies of endosperm color in sorghum proposed that the trait was oligogenic [49,50]. To date, a single gene (*Rc*) has been verified to be responsible for pericarp pigmentation of rice grains (Figure 4a), while gene *Rd* is assumed to play a role in spreading pigment [51,52]. In view of the similar levels of gene duplication in rice and sorghum, it is plausible that pericarp color is also an oligogenic trait in sorghum. In sorghum, it is known that the gene *yellow seed1* (*y1*) is required for the production of red phlobaphene pigments in the grain pericarp [46,47], and the *Tan1* gene controls tannin biosynthesis to affect pericarp color [48]. In order to minimize the limitations of artificial descriptions of colors, we applied two commonly used color models, RGB and CIE-L*ab (See the Methods also for the details), to phenotype pericarp color of sorghum seeds. On chromosome Sb01, an association peak (~61.45 Mb) in a hotspot (58 Mb-67 Mb) for red, green and blue in RGB measurement, and the values of ‘L’ and ‘a’ in the Lab model (Additional file 2: Figure S6), is close to *y1* (~61 Mb). An additional association peak (~61.86 Mb) near *Tannin1* (*Tan1*) (~61.6 Mb), is localized in a hotspot 57.5 Mb-62 Mb on chromosome Sb04 to be associated with red and green in the RGB and ‘L’ in the Lab.

Dry inflorescence weight, inflorescence length/width, and whorl number, were each very sensitive to the environment, with no colocalized hotspots between the two years for which we have data. The most striking ‘skyline’ determined by GWAS for dry inflorescence weight is centered at ~57 Mb on chromosome Sb09 (Additional file 2: Figure S7), which is also associated with plant height and flowering time. For inflorescence length the most striking association hotspot centered at ~45 Mb on chromosome Sb06 (Additional file 2: Figure S8), a location also connected with plant height and flowering.

To further investigate the inference that guinea and durra may have experienced independent domestication events but achieved non-shattering by convergence, we examined two-locus haplotypes for loci associated with pericarp color and awn presence respectively, both considered to be traits that may be subject to selection. For pericarp color, the association peak (S1_61453639) and 7 linked (*r*^2^ ≥ 0.6) SNPs in the hotspot (chromosome Sb01: 57,017,032 bp-68,413,103 bp) are used to calculate the ratio of common haplotypes/total haplotypes for each pair of sorghum types (See the “Phylogenetic relationships of five main sorghum races” also for the details). Similarly, we used an association peak (S3_72702502) and 18 linked SNPs in the hotspot (chromosome Sb03: 68,912,931 bp-74,241,979 bp) to calculate two-locus haplotype ratios for awn presence. Compared to other sorghum race pairs, guinea-durra only shows modest frequencies of common two-locus haplotypes for pericarp color (freq = 0.22) (Additional file 2: Figure S18a) and awn presence (freq = 0.26) (Additional file 2: Figure S18b). Our findings further support the hypothesis that independent domestication of guinea and durra involved convergent selection for non-shattering.

### Race-specific patterns in awn presence variation

Geographic origins and domestication history can result in patterns of phenotypic variation among genotypes within a gene pool. We investigated whether awn presence exhibits variation patterns correlated with race-specific alleles for sorghum. The hotspot (chromosome Sb03: 68,912,931 bp-74,241,979 bp) for sorghum awn presence, with the association peak (S3_72702502), is detected by both GWAS and QTL mapping (See “Traits related to the sorghum inflorescence” also). The association peak is located at an intergenic locus, which is flanked by two annotated genes [*Sb03g045420* (chromosome Sb03: 72,681,274 bp-72,687,688 bp) (similar to Hexokinase-3) and *Sb03g045430* (chromosome Sb03: 72,703,668 bp-72,704,913 bp) (similar to Putative uncharacterized protein)], based on published reduced representation sequence [4]. In this scenario, it is probable that the causative genes/loci were not genotyped, and that S3_72702502 and the causative loci shared history of mutation and recombination.

An early study [53] described morphology of panicles for two of the five main sorghum races. We (Figure 3b) show allele distribution at the locus S3_72702502, reflecting alleles ‘T’ and ‘G’ associated with awned and awnless sorghum panicles individually, finding correlation between the alleles and the race-specific morphology of awns. Allele ‘G’ with the dominant frequency in race kafir corresponds to awnless cylindrical-shaped panicles of kafir [53]. Indeed, we found 84% of kafir accessions to have awnless panicles in our phenotypic data. Similarly, allele ‘T’ with the dominant frequency in race durra, corresponds to bearded and hairy panicles of durra [53]. The major allele in race caudatum is ‘G’, consistent with the finding that 92% of caudatum accessions for which we have data are awnless. Our findings also suggest that the two most primitive sorghum types, bicolor and guinea, derived more allele ‘G’ from wild sorghum which is dominated by more ancestral allele ‘T’.

### Traits related to plant height

We conducted GWAS for 6 traits related to sorghum plant height, including total plant height, distance from base to flag leaf (base-flag), distance from rachis to flag leaf (rachis-flag), number of nodes on the main stalk (nodes), dry stalk weight and days to flowering. Strong positive correlation (Additional file 1: Table S1) are observed among the phenotypic data of total plant height, base-flag, nodes, dry stalk weight and days to flowering. It is important to remember that 228 accessions in the sorghum association panel (SAP), are converted tropical lines that are photoperiod insensitive, early maturing, and short statured phenotypes, developed by crossing exotic lines and U.S. cultivars [14,22]. Three independent studies [4,14,54] revealed three consistent genomic regions in sorghum that contribute to the introgression of cultivar-specific alleles to exotic sorghum lines. It is also clear that *Dwarf* (*Dw*)*4* and *Dw2* are associated with the introgression region in the heterochromatin of chromosome Sb06, and *Dw3* and *Dw1* are associated with the introgression regions in the 3′ terminal euchromatin of chromosome Sb07 and Sb09 respectively.

In our studies, the *Dw1-Dw4* regions reported by Morris et al. [4] show the most striking signals associated with base-flag leaf length for two years (Additional file 2: Figure S12). Likewise, the association results of total plant height (Additional file 2: Figure S11) are consistent with that of Morris et al [4]. This suggests that environmental variables have relatively little effect on *Dw1-Dw4*.

In addition to *Dw1-Dw4*, we found noteworthy hotspot(s) for plant height located on chromosome Sb04. Our BTxSP mapping population [29] and Shiringani et al., [28] each suggested two overlapping QTL likelihood intervals on Sb04, 57.98 Mb-64.93 Mb and 48.80 Mb-58.58 Mb, to contribute to plant height (Additional file 2: Figure S11). Within both intervals, we found multiple SNPs significantly associated with base-flag and dry stalk weight (Additional file 2: Figure S12 and Figure S15). Because the terminal region of Sb04 holding these QTLs exhibited weak LD, we could not set clear boundaries for the hotspot(s). GWAS of another plant height related trait, dry stalk weight in 2009 data, also shows a clear association ‘skyline’ in the region of 48.80 Mb-64.93 Mb on Sb04 (Additional file 2: Figure S15a).

Plant height and flowering time show significant genetic correlation. In further agreement, three association loci for flowering time show strong LD with dwarfing genes, and are distributed in the introgression regions. There is some confusion concerning the identities of *Ma1* and *Ma6*, with one group suggesting that *Ma1* is a sorghum ortholog of a Triticeae flowering gene, *PRR37* [55], but which is located very near the published position of *Ma6* [45]. Many of the same authors who report that *PRR37* is *Ma1* have stated under separate cover that *PRR37* is *Ma6* [56]. The LD pattern suggests two haplotype blocks in the region of 6 Mb-46 Mb on chromosome Sb06 (Figure 2b), suggesting that *Ma1* (or *Ma6*) and *Dw4* [4] are tightly linked in the region of 6 Mb-39 Mb, and *Dw2* [4] is linked with *Ma6* (or *Ma1*) in the region of 40 Mb-46 Mb on Sb06. We also identified a haplotype block of 56 Mb-59.5 Mb on Sb09 (Figure 2c), which is strongly associated with plant height and flowering time. Greater mapping resolution is required to pinpoint the causative genetic mutation(s) that affect each trait.

### Selective signatures and phenotype-genotype association

**Figure 5:**
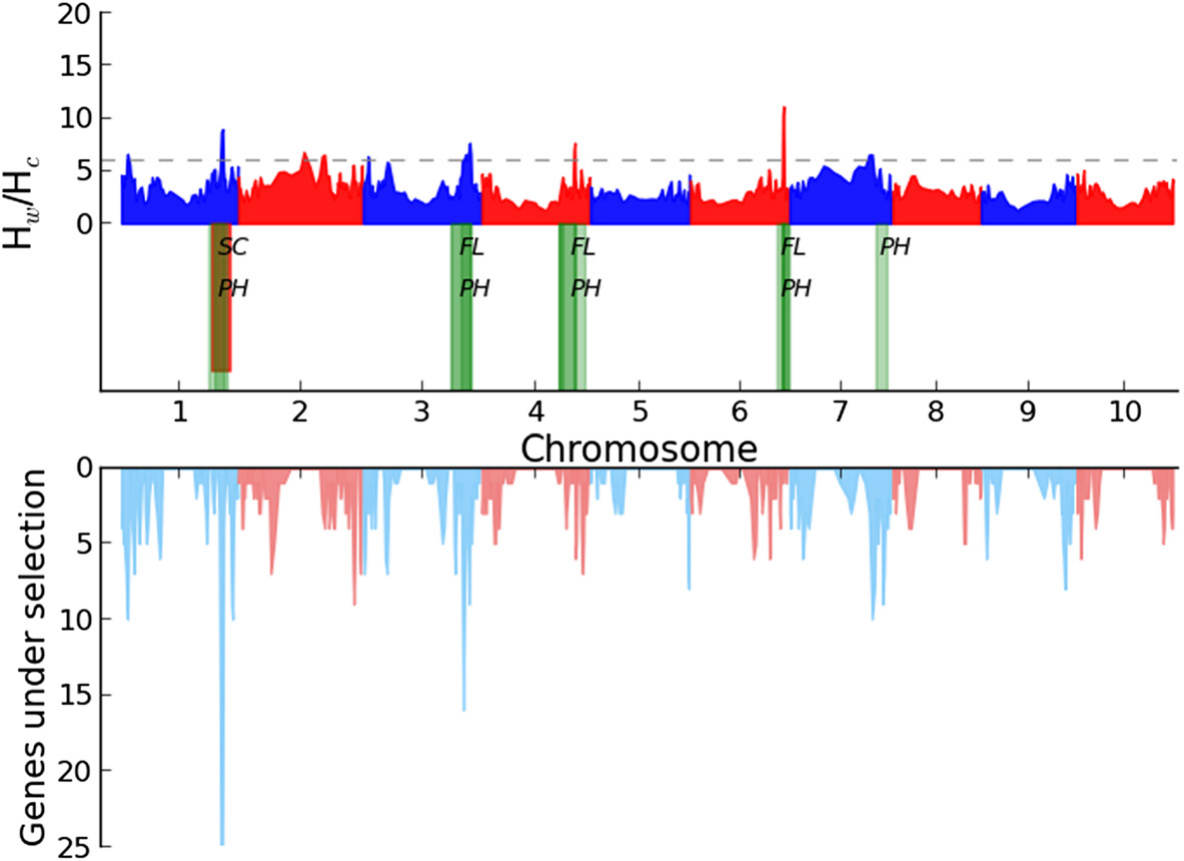
Genome-wide selection signatures. We calculated average ratios (expected heterozygosity of wild type sorghum (H_w_) / expected heterozygosity of cultivated sorghum (H_c_)) for windows of 500 consecutive SNPs throughout the genome. The gray dashed line is the cutoff for the top 5% of heterozygosity ratios. Green areas indicate confidence intervals determined by QTL mapping to contribute to either flowering time (FL) or plant height (PH), that also have strong selection signals. The hotspot on chromosome Sb01 identified by GWAS for pericarp color (SC) is highlighted by red. The distribution of 725 candidate genes implicated in domestication and/or improvement via gene-based population summary statistics [41] is shown below the heterozygosity ratios.

A phenotype-genotype association is no guarantee that the trait or its candidate gene has been historically important or is an adaptation [57]. Reduction in genetic diversity, which can be assessed by heterozygosity, can bear the signature of domestication [4,58,59]. Using quantified genome-wide heterozygosity, low heterozygosity regions in sorghum have been reported [4] but have not shown significant correspondence with identified loci associated with plant height, one of the key domestication traits. Here, we refined analysis of selective signatures from domestication and explored the genomic regions that have been important in sorghum adaptation for inflorescence morphology and plant height. Genetic diversity of the sorghum population was assessed by the ratio of expected heterozygosity in wild sorghum to that in cultivated types (H_w_/H_c_) across the sorghum genome (Figure 5). Overall, the refined pattern of reduction in genetic diversity is consistent with that measured previously [4]. Among the hotspots determined by association mapping for the 12 traits, the genomic region 58 Mb-64 Mb on chromosome Sb01 for pericarp color shows a strong selection signal based on reduction in heterozygosity ratio (Figure 4a). Such correspondence was also discovered in rice [60], consistent with the observation that pericarp color has profoundly influenced the popularity of cultivated cereal varieties [61].

Some confidence intervals identified by QTL mapping for plant height and flowering time [14,24-28] colocalized non-randomly (*p* = 0.231×10^-6^) (See the overlapping testing in the “Methods”) with regions having striking reductions in heterozygosity ratio (Figure 5). For example, Hart et al., [24] and Ritter et al., [26] each identified a region (55.1 Mb-66.7 Mb) for plant height on chromosome Sb01 which showed strong selection signals. A region of 55 Mb-62 Mb on chromosome Sb06, showing strong selection signals, was identified for plant height by Kebede et al., [25], Srinivas et al., [27] and Shiringani et al., [28], and for flowering time by Shiringani et al., [28]. Additional heterozygosity ratio peaks overlapping with plant height and flowering time intervals are found on Sb03 and Sb04. Most such colocalizing intervals affect plant height and flowering time simultaneously, and do not colocalize with hotspots determined by association mapping.

Using 8 M SNPs characterized in 44 sorghum lines, a recent study [41] implicated 725 candidate genes in sorghum domestication and/or improvement via gene-based population summary statistics. We show (Figure 5) at least two regions having striking reductions in heterozygosity ratio, to also be enriched for candidate genes under selection. One striking case is the 58 Mb-64 Mb interval on chromosome Sb01, in genetic control of pericarp color and plant height [24,26]. The other case is the 62 Mb-64 Mb interval on chromosome Sb03, in genetic control of plant height and flowering time [14,26,28].

Much of the ‘missing heritability’ from GWAS has been speculated to be attributed to the low/rare frequency of causative mutations, which are unlikely to be detected by most association studies [62]. Some genetic variations with large effect may be hidden by neutral genetic diversity in a natural population. By contrast, biparental QTL mapping uses genetic markers (e.g. SSR and RFLP), and is often derived from parental lines that differ strongly in phenotype such as our *S. bicolor* × *S. propinquum* cross between parents which are separated by 1-2 million years. Sometimes, biparental QTL mapping can be more efficient than GWAS to detect alleles that are rare in populations. QTL mapping and GWAS are thus two complementary approaches to determine “saturation” in terms of QTL discovery. Our findings lead to the prediction that the likelihood intervals on chromosome Sb01, Sb03, Sb04 and Sb06 identified by QTL mapping should bear the signature of selection, and may encode important variants with low/rare frequency for plant height and flowering time.

### Genetic correspondence between sorghum and rice

Synteny and colinearity have been well conserved between grass species such as rice, maize, and sorghum since their divergence about 50 Mya [21], enabling us to compare causal loci in corresponding regions across taxa. SSR mapping in rice revealed that two intervals affecting awns are located on chromosome Os01, and designated as *An9* and *An10*, respectively (Figure 4b) [63]. *An10* produced shorter and sparser awns than *An9*, and is thought to be more important for awn presence in rice. Recently, a rice gene, *OsETT2*, which colocalizes with *An10*, has been identified using genetic analyses and RNA-silencing experiments, to be involved in awn formation [64]. Chromosome Sb03 harbors the most significant hotspot for awn presence in sorghum, bounded in a region sharing large-scale homoeology with a region on Os01 containing *An10* and *OsETT2* (Figure 4b). The two hotspots do not appear to have direct correspondence across taxa, making it unclear whether there has been some localized rearrangement, functional divergence of different members of a co-regulated cluster of genes, or some other explanation.

Duplication and subsequent rearrangements can result in chromosomes exhibiting a complex pattern of homoeology across taxa. The *Rc* gene is responsible for conditioning red pericarp in rice [52]. Association mapping shows no noteworthy signal around the sorghum ortholog (*Sb02g006380*) of *Rc*, or for another sorghum gene copy (*Sb01g028230*). Rearrangement following polyploidization can lead to a scrambled patchwork in genome organization [65]. In the case of pericarp color, the correspondence of gene arrangement between the most significant hotspot in sorghum and the rice *Rc* region is still discernible through multiple alignment (Figure 4a). The hotspot on sorghum chromosome Sb01 shares homoeology with three small genomic segments on Sb01, one of which has correspondence with the region enclosing *Sb02g006380* on sorghum chromosome Sb02. Thus, despite discernible parallels in genome organization, corresponding genes do not appear to be responsible for phenotypic variation in seed color of sorghum and rice. This may simply reflect that 40-50 My of rice-sorghum divergence, did involve many genetic changes that were different, as well as some that may have been convergent [18].

Both linkage studies and reductions in heterozygosity ratios (Figure 4c and d) implicate two genomic regions in genetic control of plant height and flowering time, which are located in the 58 Mb-64 Mb on chromosome Sb01 [24,26] and in the 62 Mb-64 Mb on chromosome Sb03 [14,26,28] individually. In rice, gene *Osg1* [66], which is located on chromosome Os01 (Figure 4c) orthologus to sorghum chromosome Sb03, has been known to affect plant height via reduction in its expression; genes *Ehd4* [67] and *Hd16* [68] on chromosome Os03 orthologous to sorghum chromosome Sb01, are characterized by their function in flowering time of rice. We observed that sorghum has experienced gene loss for *Ehd4*, and none of three rice genes shows direct correspondence with the two genomic regions in sorghum. These findings indicate that different genes, rather than orthologs of *Osg1*, *Ehd4* and *Hd16*, were identified by QTL mapping on Sb03 and Sb01.

### QTL correspondence across homoeologous regions within sorghum

QTLs may share homoeology not only across taxa, but also within taxa. Maize, in which many homoeologous chromosome segments have been identified as a result of lineage-specific genome duplication, is a particularly favorable organism in which to show such QTL correspondence [14]. Only occasional cases have been found in sorghum [14], probably due to much greater antiquity of genome duplication [1].

**Figure 6:**
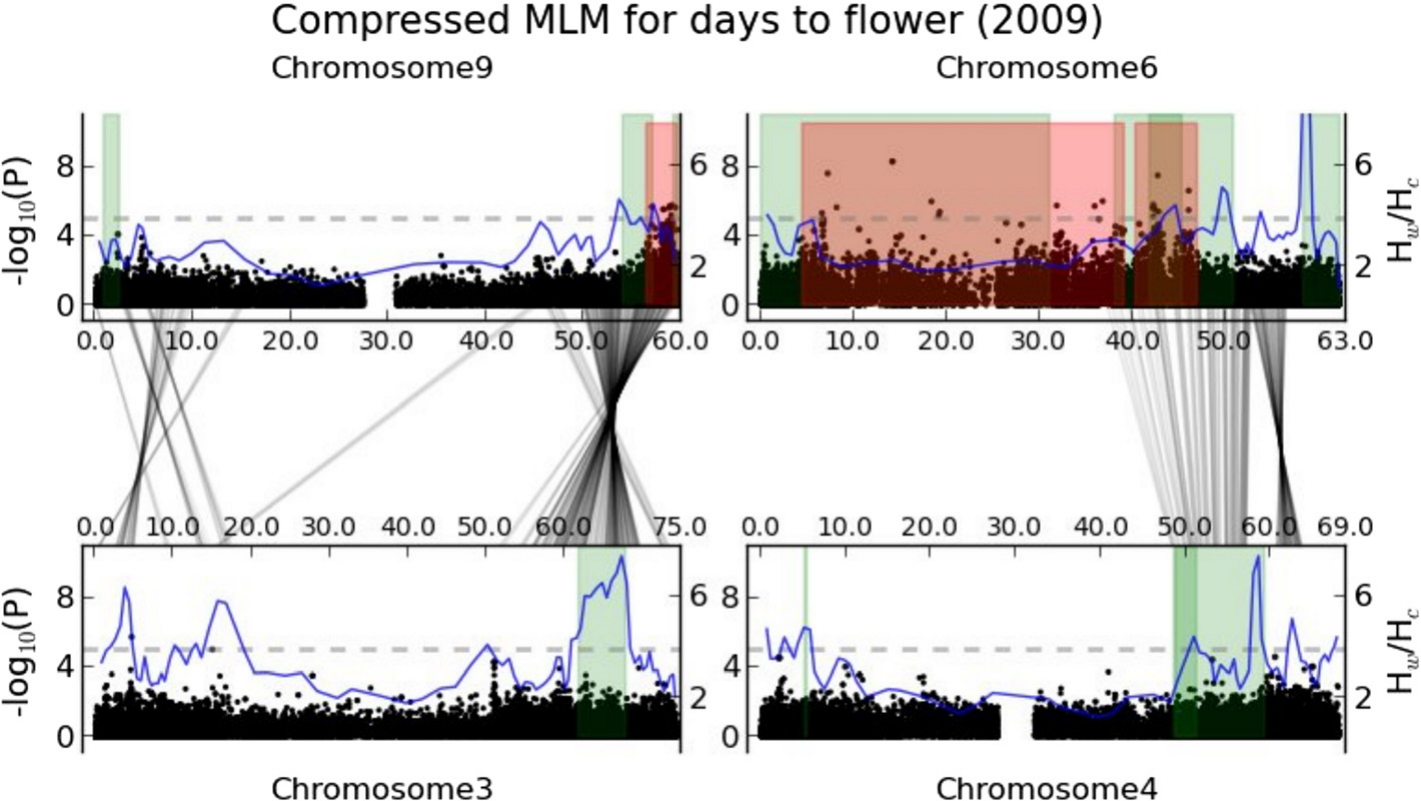
Genetic correspondence within taxa. Intra-genomic genetic correspondence for QTL intervals for flowering time on chromosome pairs of Sb03-Sb09 and Sb04-Sb06.

A total of 4 pairs of homoeologous QTLs for plant height/flowering time are found in our study. For flowering time, we observed two instances of homoeologous QTLs in sorghum (Figure 6). The confidence interval mapped on chromosome Sb03 [28] shares homoeology with the hotspot of 54 Mb-59.6 Mb on chromosome Sb09 which has been identified by QTL mapping [23] and our GWAS. The other correspondence was determined between 48.6 Mb-59.3 Mb on chromosome Sb04 [26,28] and 38 Mb-50.7 Mb on chromosome Sb06 [14,18]. Likewise, we found homoeologous QTLs for plant height on chromosome pairs of Sb03-Sb09 and Sb04-Sb06 (Additional file 2: Figure S17). The additional cases for flowering time are shown on chromosome pairs of Sb02-Sb07 and Sb05-Sb08 (Additional file 2: Figure S17). Such genetic correspondence within sorghum implies that some homoeologous regions created in a genome duplication 70 million years ago [21] may still retain some genes with similar functions. In most instances, we found that one QTL hotspot exhibits much stronger selection signal than its homoeologous counterpart on the basis of genetic diversity reduction in the wild population. Independent episodes of domestication and crop improvement may preferentially select one counterpart, but may or may not select the other.

## Conclusions

Our studies illustrate how GWAS may be used to complement the genetic resolution of causal elements (genes) of quantitative phenotypes that is typically attained from conventional likelihood intervals determined by QTL mapping. The degree of improvement in resolution by GWAS over QTL mapping is related to the nature of the ‘genomic environment’ surrounding a gene – with substantial improvement in recombinationally-active euchromatin but much less improvement in recombinationally recalcitrant heterochromatin with long LD blocks.

Understanding and utilizing the relative strengths and weaknesses of QTL mapping and GWAS can aid in dissecting the genetic basis of a complex trait. A carefully chosen cross can allow QTL mapping to have better statistical power to detect variants with low/rare frequency in a natural population. For example, the classical maturity locus *Ma3/phyB* was initially identified with a map-based strategy (QTL mapping) [69], but association mapping is unable to detect striking signal near *Ma3* because virtually all members of the panel are wild type (i.e. the mutation is rare). Another case is that a map-based (QTL mapping) strategy was used to determine one major-effect QTL (*sh1*) controlling shattering in sorghum, and revealed three different non-shattering haplotypes widely existing in five major cultivated races that may reflect low/rare frequency of causative polymorphism [2,3]. Hence, association mapping may have reduced power to find phenotype-genotype association for *sh1*. Additionally, complex population structure in unrelated individuals may compromise genetic variation at the true causative loci, resulting in cases of true negative detection in GWAS. More generally, multiple independent domestications may create a scenario under which many QTLs cannot be verified with the current sorghum association panel.

**Figure 7:**
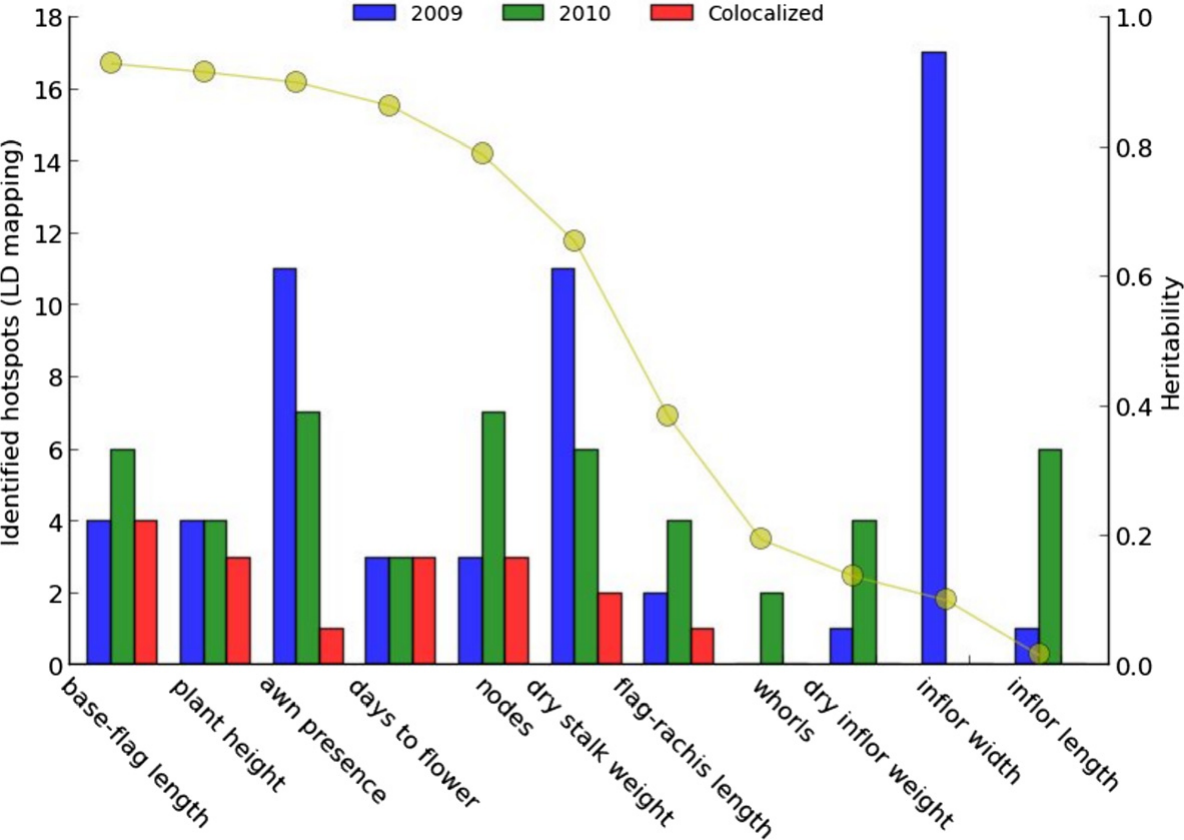
Summary of hotspots identified by GWAS for multi-year trait data. For each trait, numbers of hotspots for 2009, 2010 and both years are indicated with bars. The values of heritability for the traits are indicated with the curve.

An important fundamental question in gene mapping is why the GWAS approach has revealed so much less variation than anticipated [62]. Even given ‘perfect’ (i.e. 100% accurate) information about phenotype and genotype, some associations may not be repeatable due to interaction between genotype and environment [57]. We observed that traits related to the inflorescence were more greatly affected by environment than traits related to plant height (Figure 7). The interaction between genotype and environment plays an important role in the variation between individuals in their observable characteristics, even for traits with high heritability.

Identification of intersections among QTL, GWAS and comparative data advance knowledge of the genetic determinants of variation in sorghum, and provide a finer-scale comparison than was previously possible of the genetics of important traits between sorghum and rice. Non-random correspondence of sorghum GWAS signals to rice QTLs, i.e. between divergent panicoid and oryzoid grasses, adds a new dimension to evidence of the ability to leverage genetic data about the important traits across divergent plants. More variants can be gained through re-sequencing gene candidates. Joint evidence from hotspots and re-sequencing data are likely to be extremely powerful to answer the question of whether such genetic correspondence implicates ‘conserved’ single genes or co-regulated clusters of genes that exist for the trait of interest after cereal divergence.

Rich SNP data also clarifies the phylogenetic relatedness of sorghum races, improving on several prior studies [38-41]. Based on the high-density genotype in the large population, we showed joint evidence from the striking clustering patterns in population structure and fixation index values, suggesting several hypotheses that might shed light on the unknown ancestry of sorghum races.

### Availability of supporting data

In addition to the supplementary files listed below, Newick files for all the phylogenetic trees are available for download at Dryad (http://datadryad.org/).

## Additional files

Additional file 1:
**The file contains supplementary Tables S1, S2 and S3.** (**Table S1.** Phenotypic correlation determined by Pearson correlation coefficient). (**Table S2.** 1-LOD likelihood intervals determined by QTL mapping. Trait names, genomic positions and references are indicated). (**Table S3.** Hotspots identified by GWAS. Trait names, years, genomic positions are indicated). Additional file 2:
**The file contains supplementary Figures S1-S18.**

## Abbreviations

QTL: Quantitative trait locus
GWAS: Genome-wide association study
SNP: Single nucleotide polymorphism
SAP: Sorghum association panel
PCA: Principal component analysis
LD: Linkage disequilibrium
MAF: Minor allele frequency
F_ST_: Fixation index
WGD: Whole-genome duplication
GBS: Genotyping-by-sequencing
CMLM: Compressed mixed linear model
