# Supplementary material for: Genetic analysis of inflorescence and plant height components in sorghum (Panicoidae) and comparative genetics with rice (Oryzoidae)
Source: BMC Plant Biol. 2015 Apr 19;15:107. doi: 10.1186/s12870-015-0477-6 (PMC4404672; doi:10.1186/s12870-015-0477-6)
Supplement: Additional file 2: — The file contains supplementary Figures S1-S18. [file 12870_2015_477_MOESM2_ESM.pdf]

Comparative genetics of inflorescence and plant height components in divergent cereal lineages represented by sorghum (Panicoidae) and rice (Oryzoidae)

Dong Zhang[1,2], Wenqian Kong[1,3], Jon Robertson[1], Valorie H. Goff[1], Ethan Epps[1], Alexandra Kerr[1], Gabriel Mills[1], Jay Cromwell[1], Yelena Lugin[1], Christine Phillips[1], Andrew H. Paterson[1,2,3,4,5,*]

[1] Plant Genome Mapping Laboratory, University of Georgia, Athens, GA 30602, USA

[2] Institute of Bioinformatics, University of Georgia, Athens, GA 30602, USA

[3] Department of Crop and Soil Sciences and Department of Genetics, University of Georgia, Athens, GA 30602, USA

[4] Department of Plant Biology, University of Georgia, Athens, GA 30602, USA

[5] Department of Genetics, University of Georgia, Athens, GA 30602, USA

FOOTNOTES

* To whom correspondence should be addressed.

Tel: +1 706 583 0162

Fax: +1 706 583 0160

Email: paterson@uga.edu

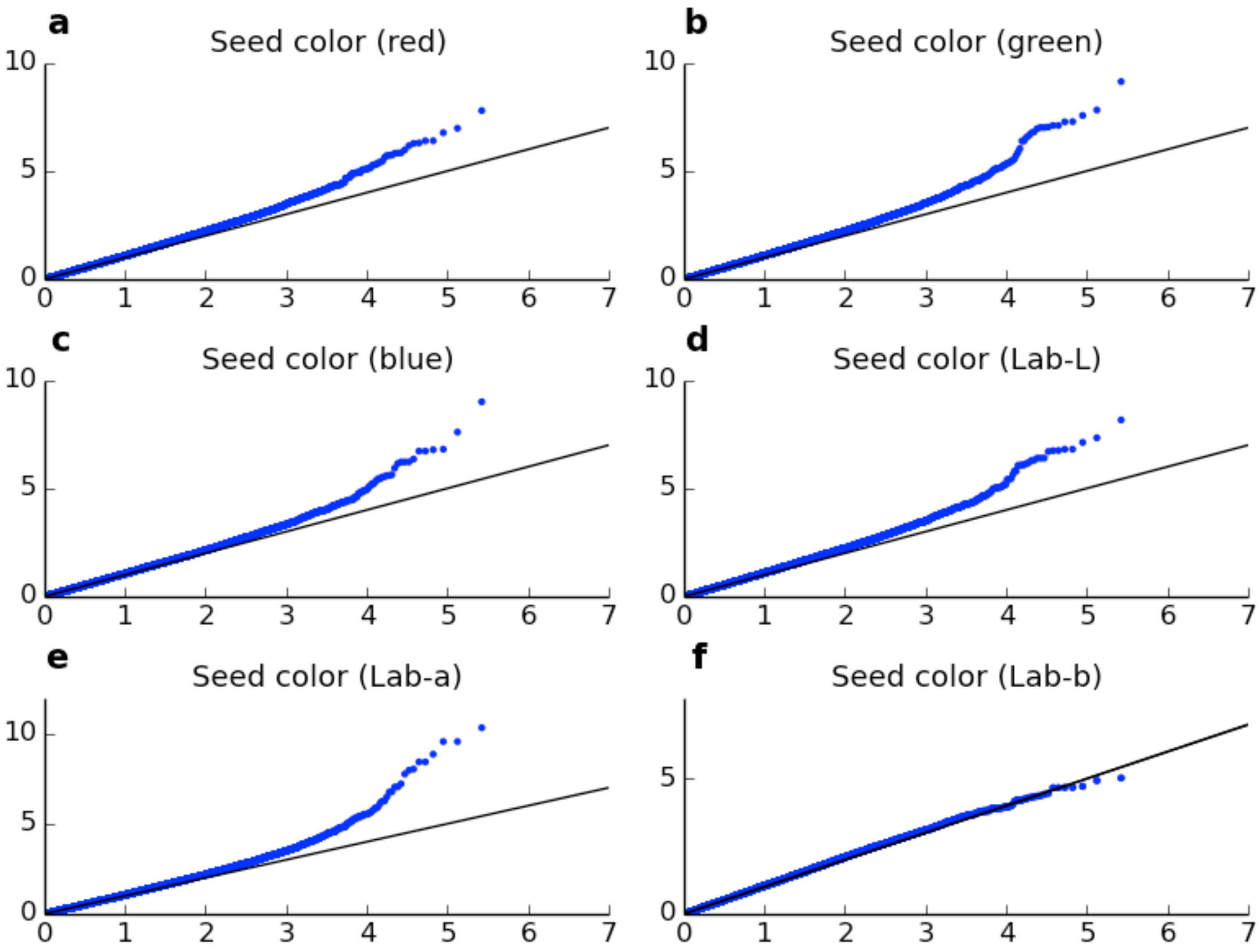

Fig. S1 Log quantile–quantile (QQ) P-value (compressed MLM) plots for 265,487 single-SNP tests of association in seed color traits. The association P values are indicated by blue lines. The black lines correspond to the null hypotheses.

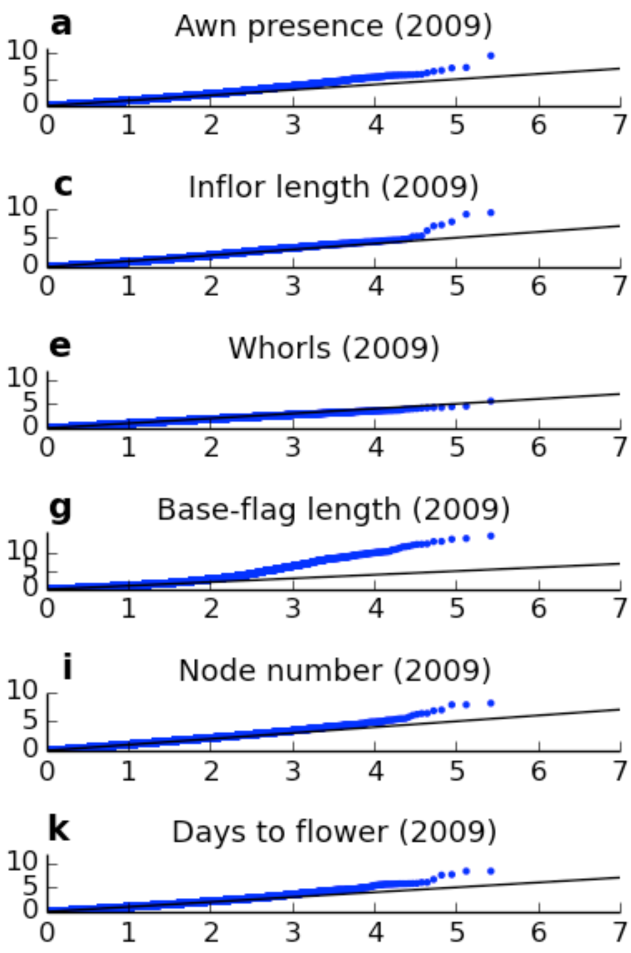

Fig. S2 Log quantile–quantile (QQ) P-value (compressed MLM) plots for 265,487 single-SNP tests of association in 11 traits measured in 2009. The association P values are indicated by blue lines. The black lines correspond to the null hypotheses.

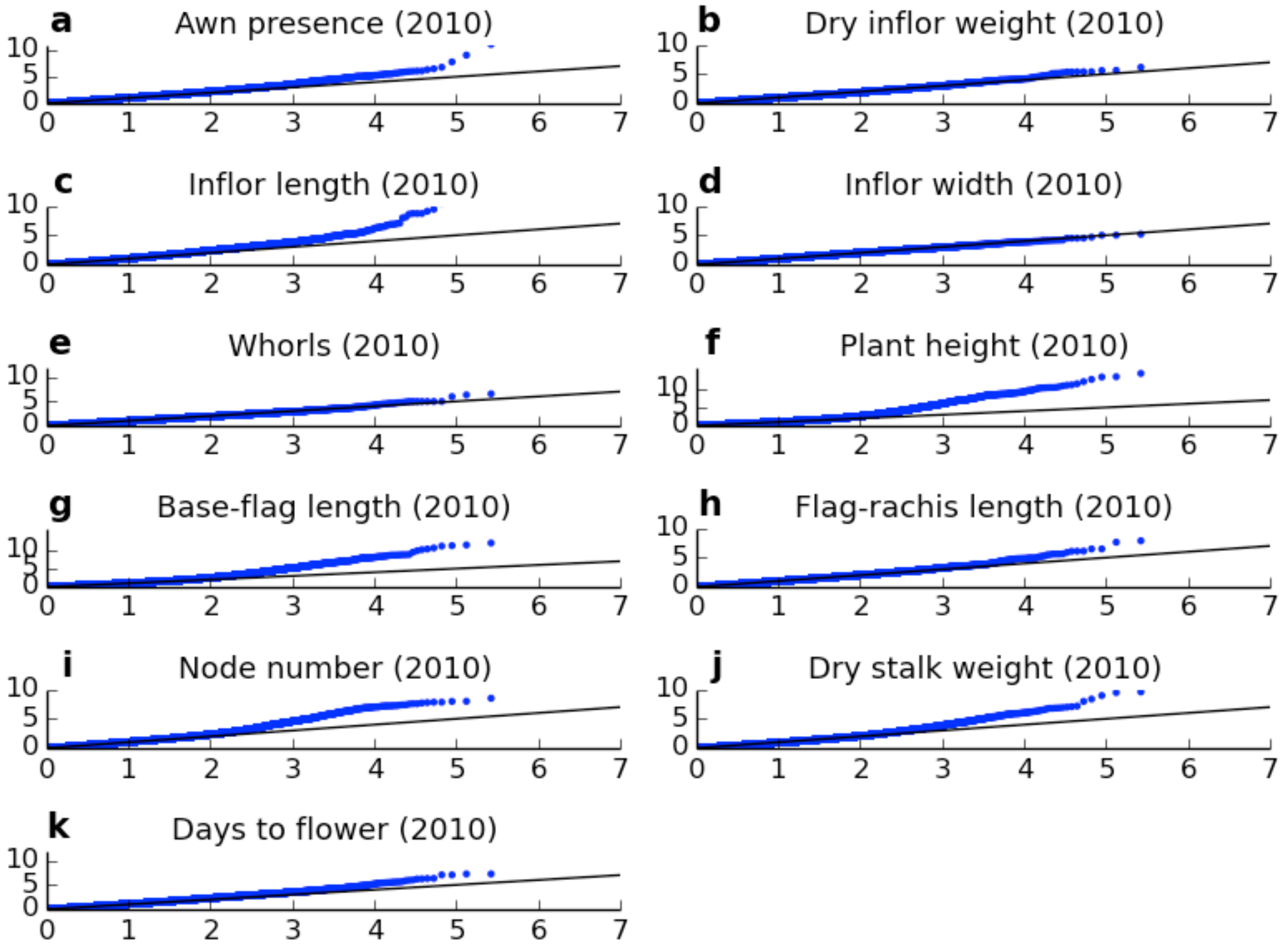

Fig. S3 Log quantile–quantile (QQ) P-value (compressed MLM) plots for 265,487 single-SNP tests of association in 11 traits measured in 2010. The association P values are indicated by blue lines. The black lines correspond to the null hypotheses.

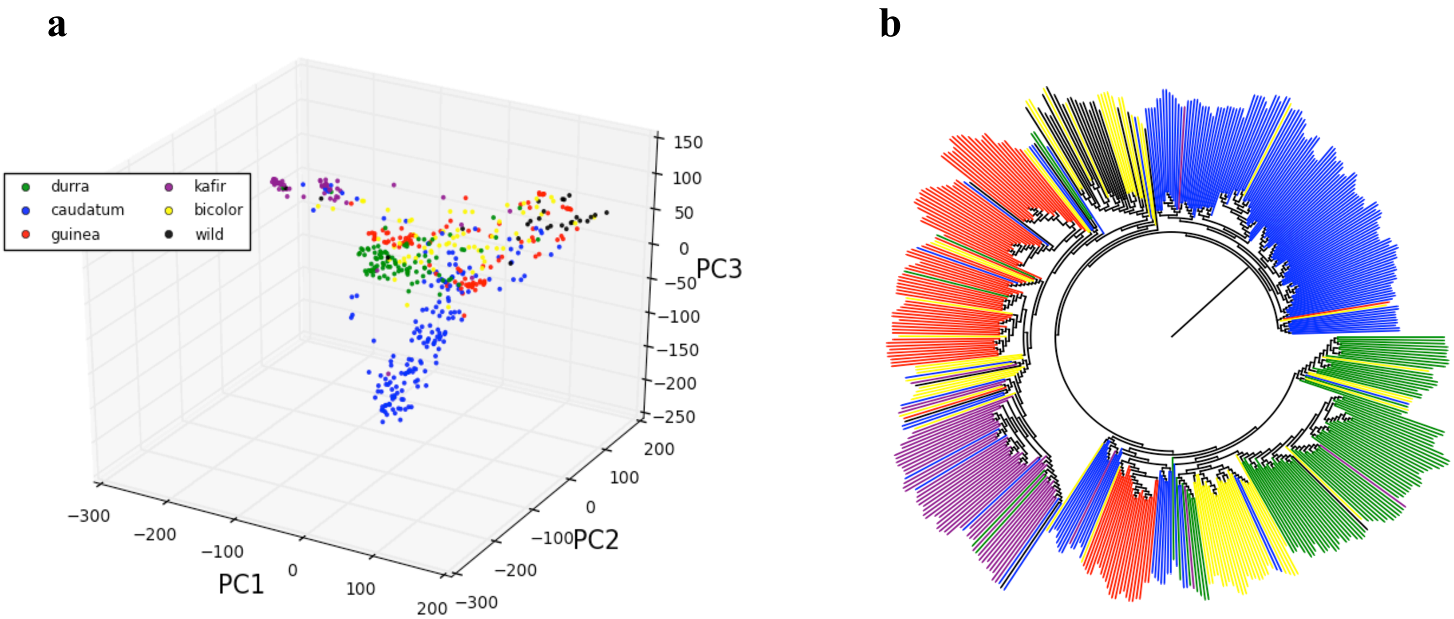

Fig. S4 Population structure of 502 worldwide sorghum accessions. 476 belong to the five main cultivated races and 26 are wild types. (a) PCA plots of the first three components for 194,852 SNPs with MAF>=0.02 and missing rate<=50%. The five main cultivated races and the wild type are color-coded. (b) Neighbor-joining tree of 502 sorghum accessions.

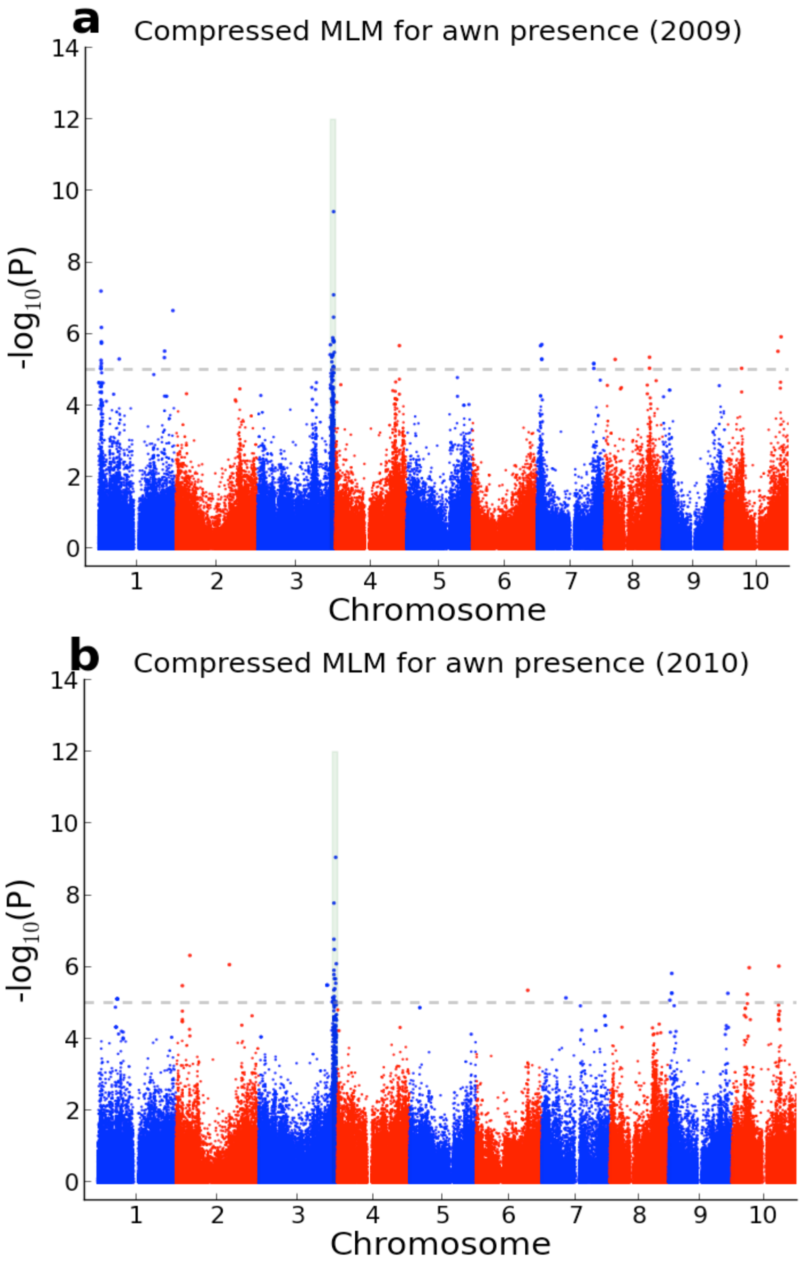

Fig. S5 GWAS for awn presence. (a) Genome-wide Manhattan plot of CMLM for 2009. Significance threshold is denoted by the gray dashed line. The 10 sorghum chromosomes are plotted against the negative base-10 logarithm of the association P value. Areas highlighted in green indicate confidence intervals for awn presence determined by QTL mapping. (b) Genome-wide Manhattan plot of CMLM for 2010.

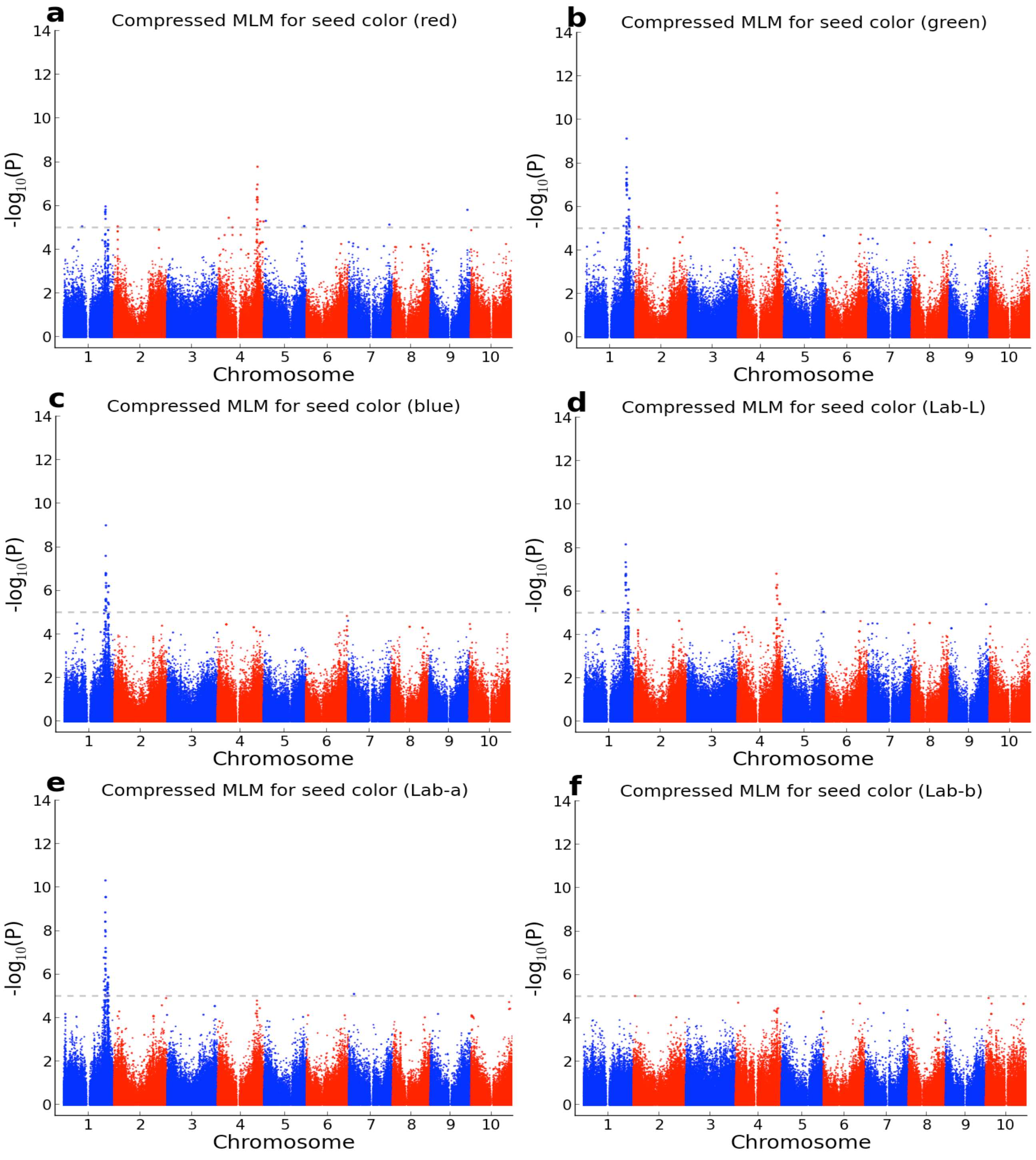

Fig. S6 GWAS for pericarp color. (a) Genome-wide Manhattan plot of CMLM for RGB-R. (b) Genome-wide Manhattan plot of CMLM for RGB-G. (c) Genome-wide Manhattan plot of CMLM for RGB-B. (d) Genome-wide Manhattan plot of CMLM for Lab-L. (e) Genome-wide Manhattan plot of CMLM for Lab-a. (f) Genome-wide Manhattan plot of CMLM for Lab-b.

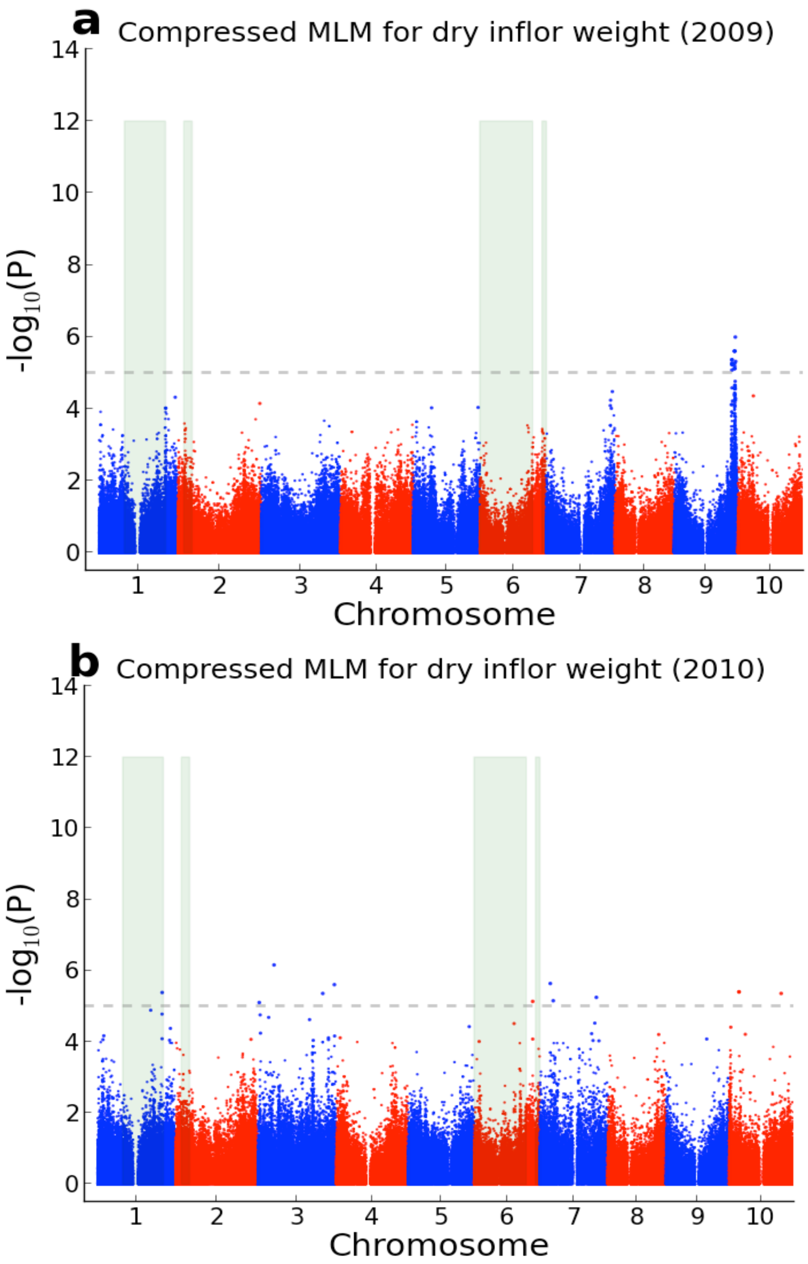

Fig. S7 GWAS for dry inflorescence weight. (a) Genome-wide Manhattan plot of CMLM for 2009. (b) Genome-wide Manhattan plot of CMLM for 2010.

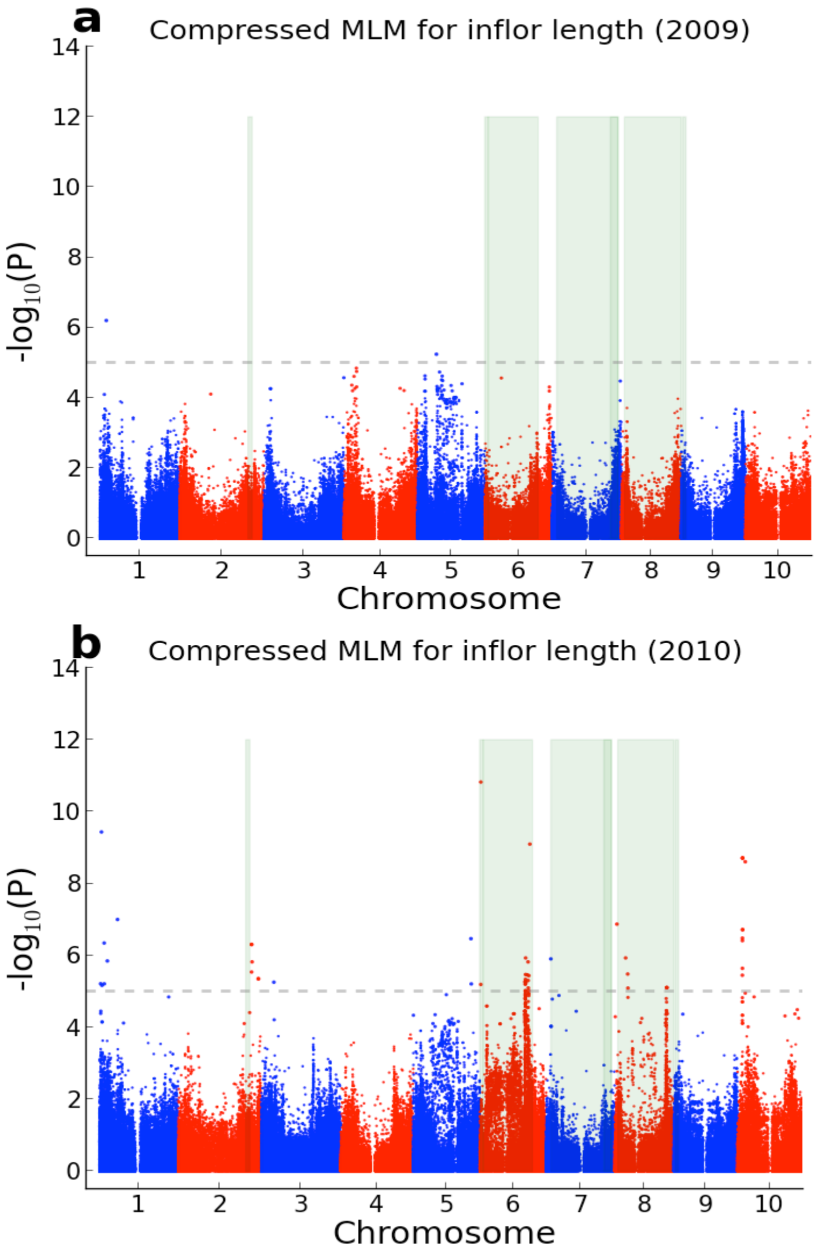

Fig. S8 GWAS for inflorescence length. (a) Genome-wide Manhattan plot of CMLM for 2009. (b) Genome-wide Manhattan plot of CMLM for 2010.

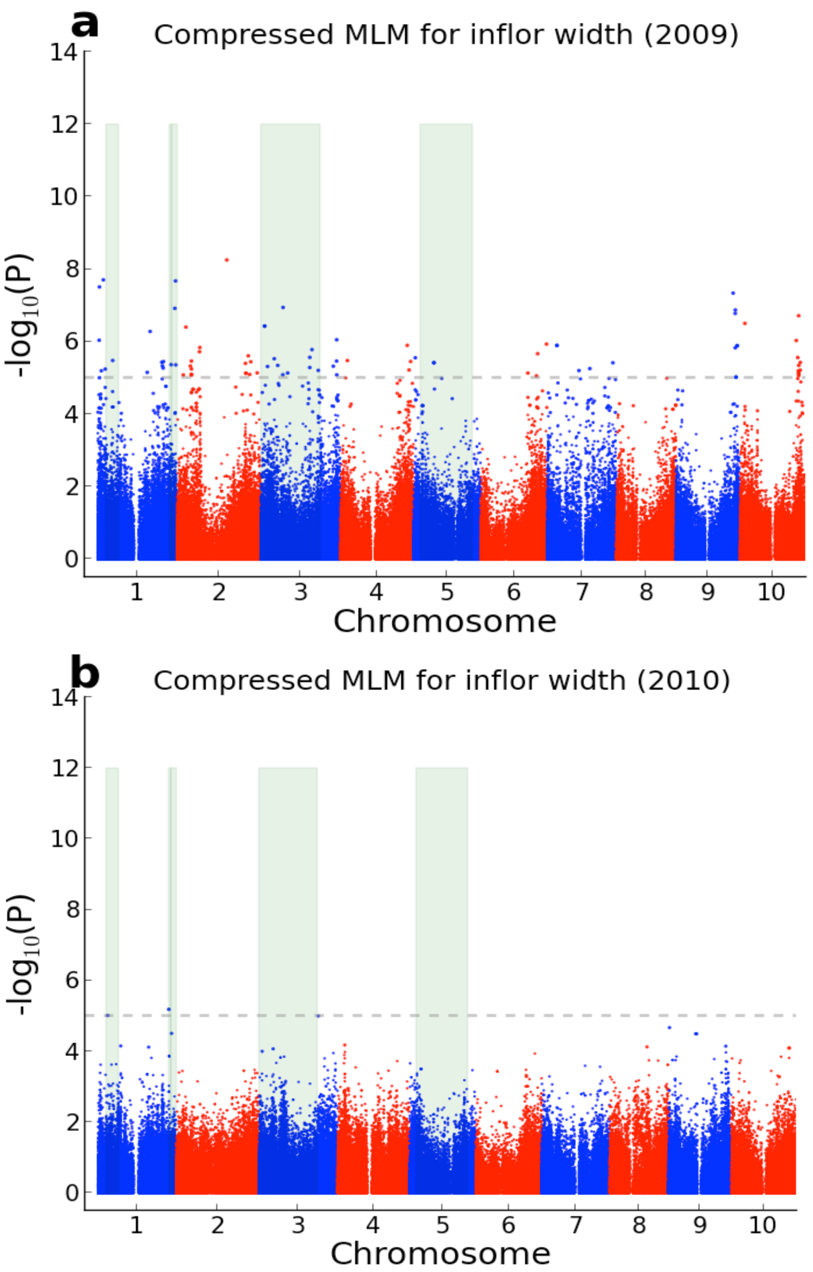

Fig. S9 GWAS for inflorescence width. (a) Genome-wide Manhattan plot of CMLM for 2009. (b) Genome-wide Manhattan plot of CMLM for 2010.

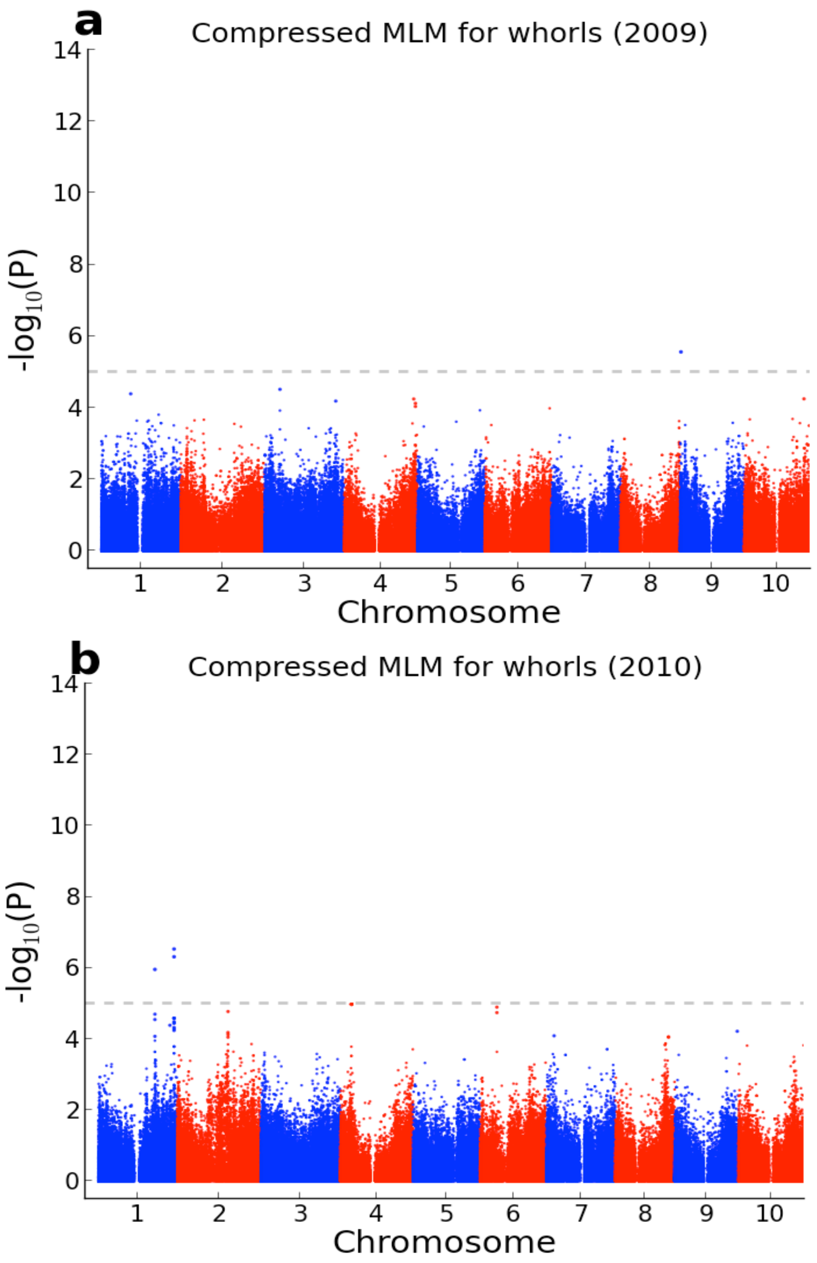

Fig. S10 GWAS for whorl number. (a) Genome-wide Manhattan plot of CMLM for 2009. (b) Genome-wide Manhattan plot of CMLM for 2010.

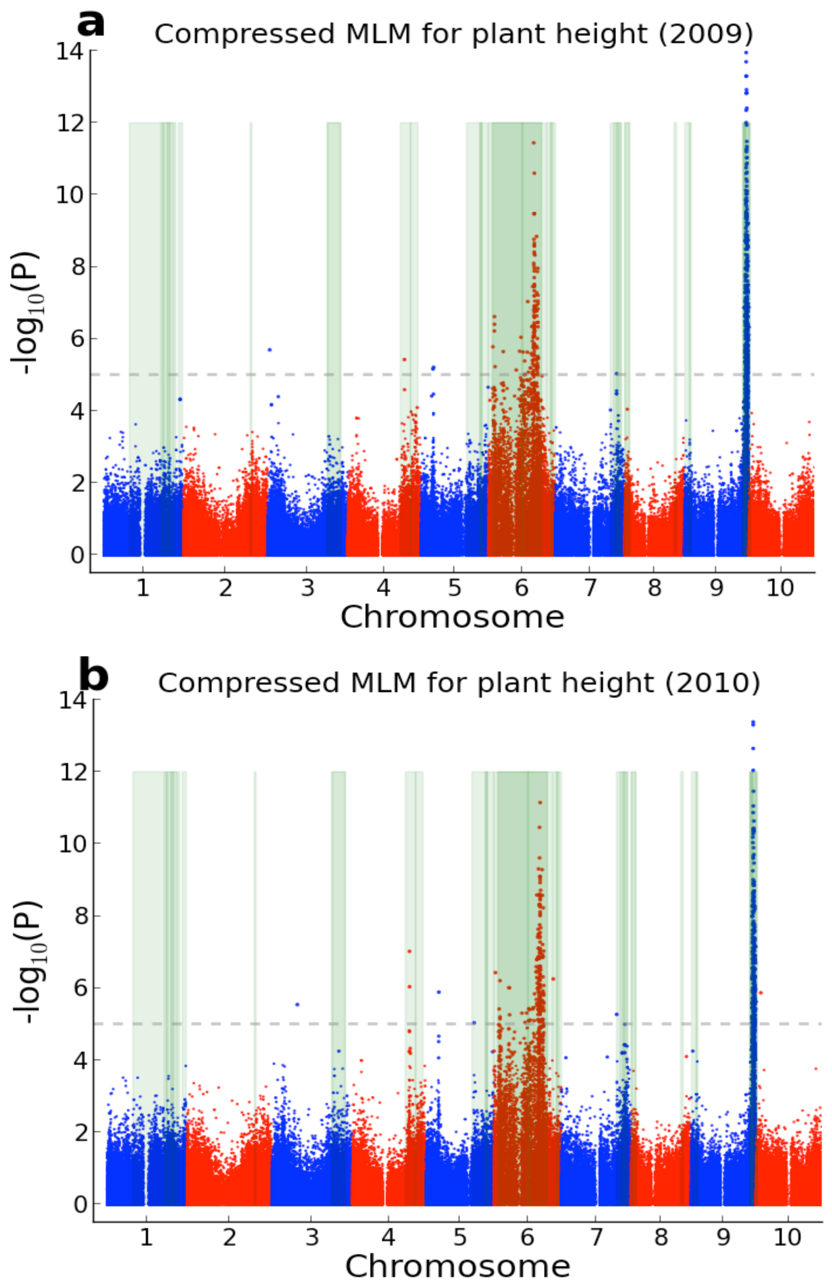

Fig. S11 GWAS for total plant height. (a) Genome-wide Manhattan plot of CMLM for 2009. (b) Genome-wide Manhattan plot of CMLM for 2010.

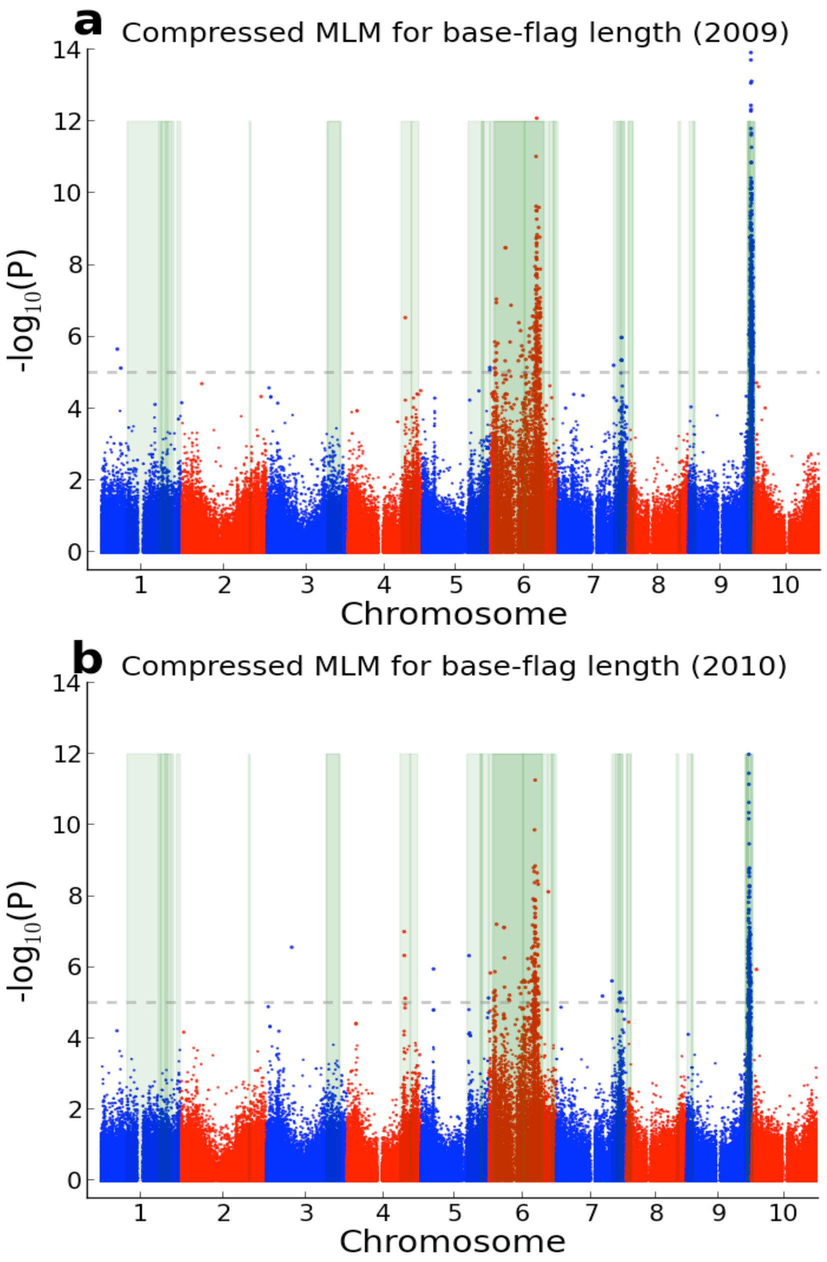

Fig. S12 GWAS for base-flag length. (a) Genome-wide Manhattan plot of CMLM for 2009. (b) Genome-wide Manhattan plot of CMLM for 2010.

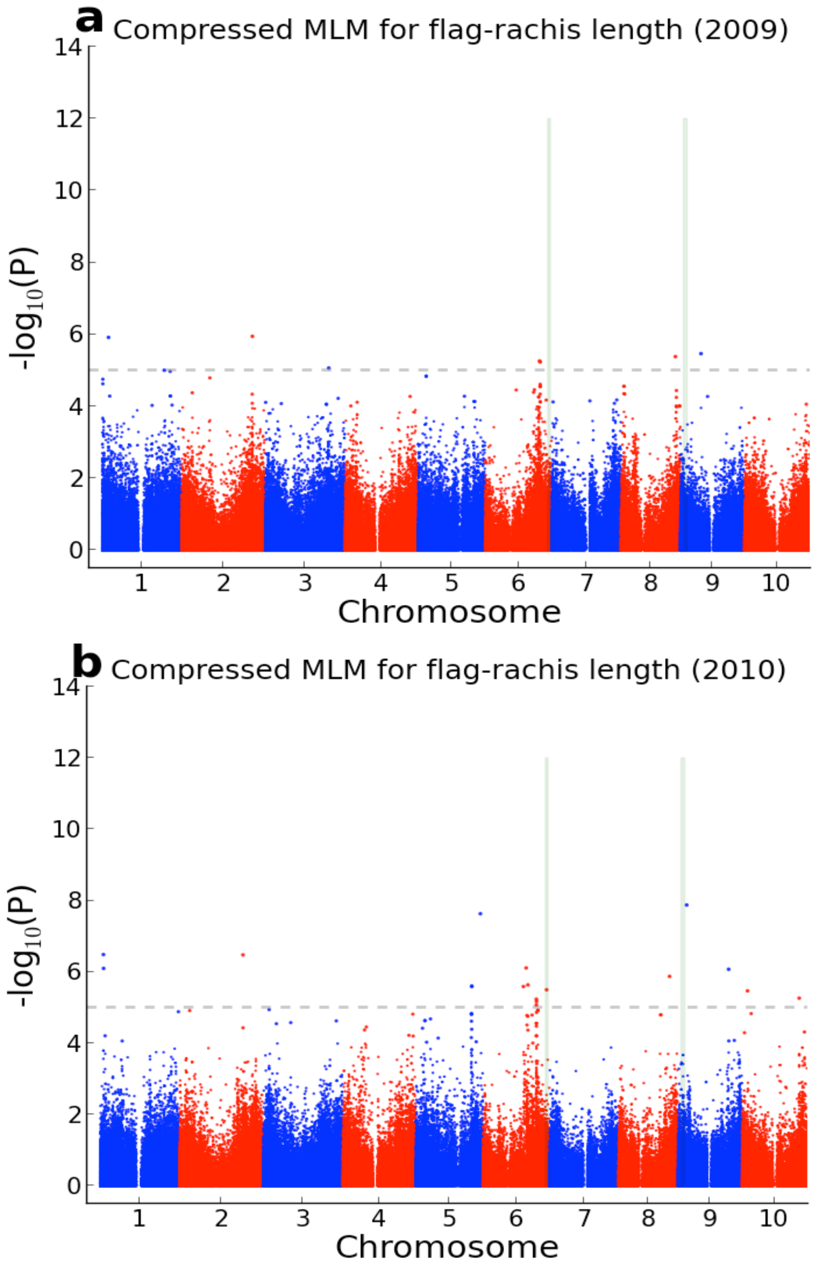

Fig. S13 GWAS for flag-rachis length. (a) Genome-wide Manhattan plot of CMLM for 2009. (b) Genome-wide Manhattan plot of CMLM for 2010.

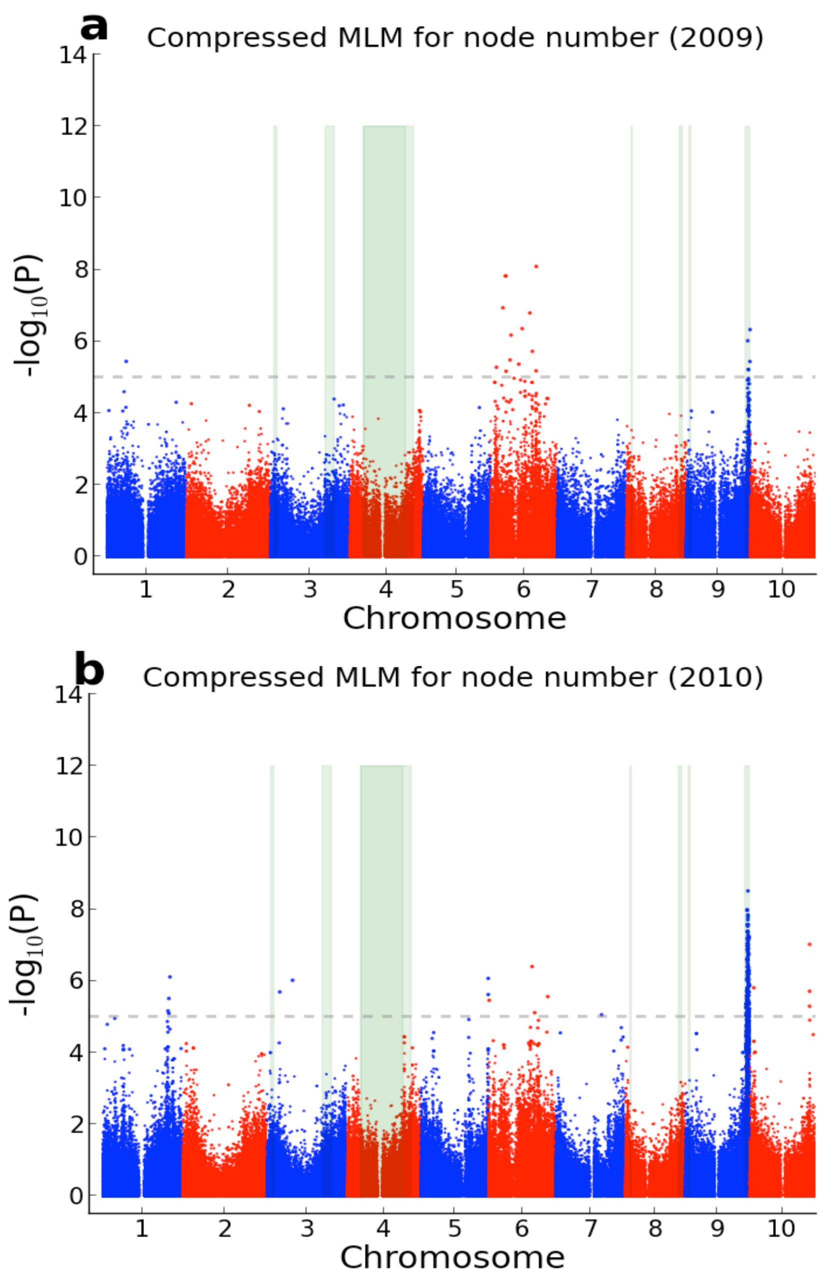

Fig. S14 GWAS for node number. (a) Genome-wide Manhattan plot of CMLM for 2009. (b) Genome-wide Manhattan plot of CMLM for 2010.

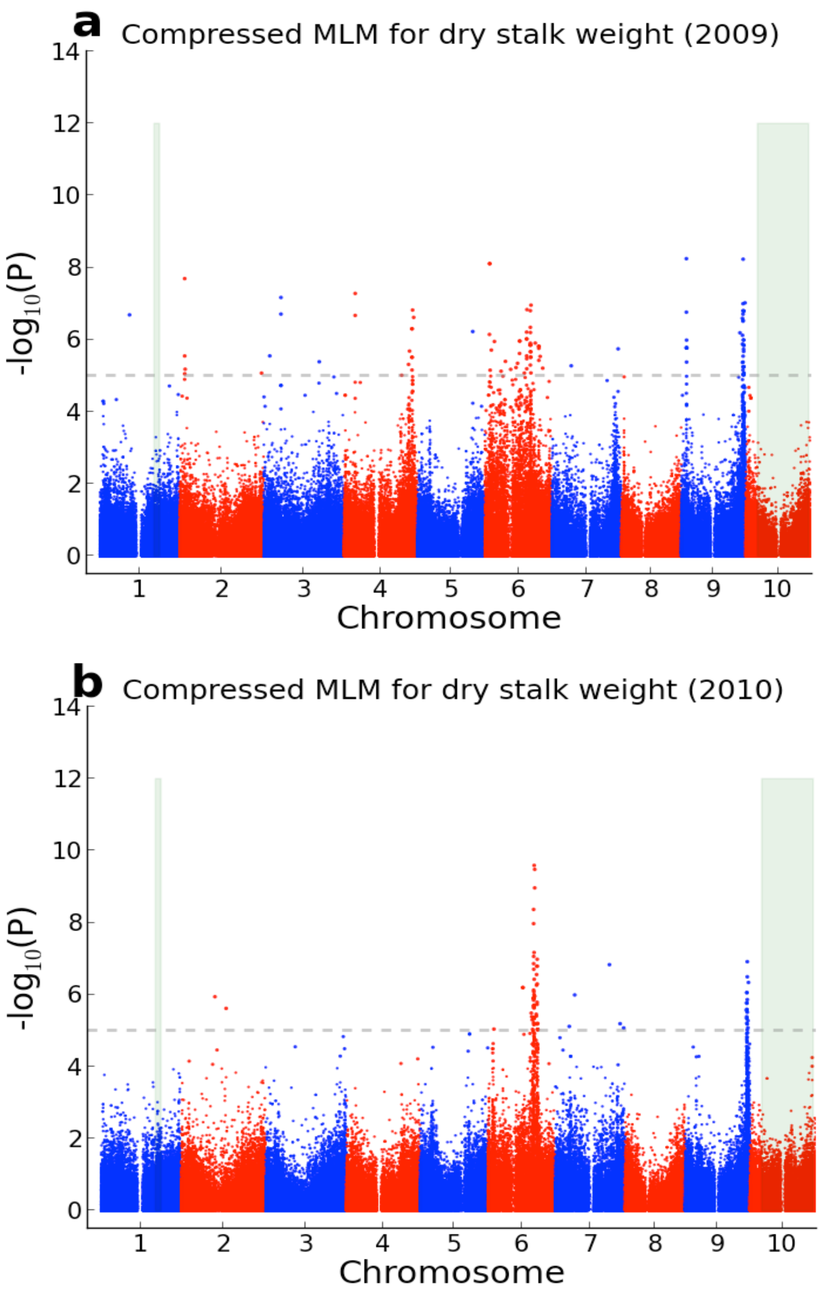

Fig. S15 GWAS for dry stalk weight. (a) Genome-wide Manhattan plot of CMLM for 2009. (b) Genome-wide Manhattan plot of CMLM for 2010.

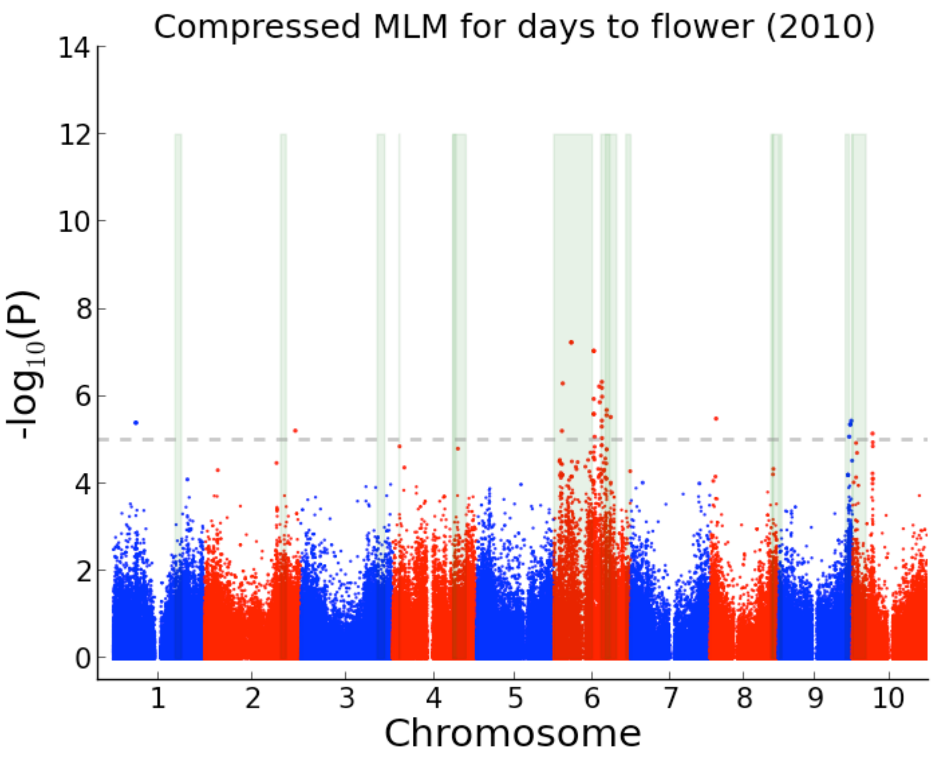

Fig. S16 Genome-wide Manhattan plot of CMLM for flowering time in 2010.

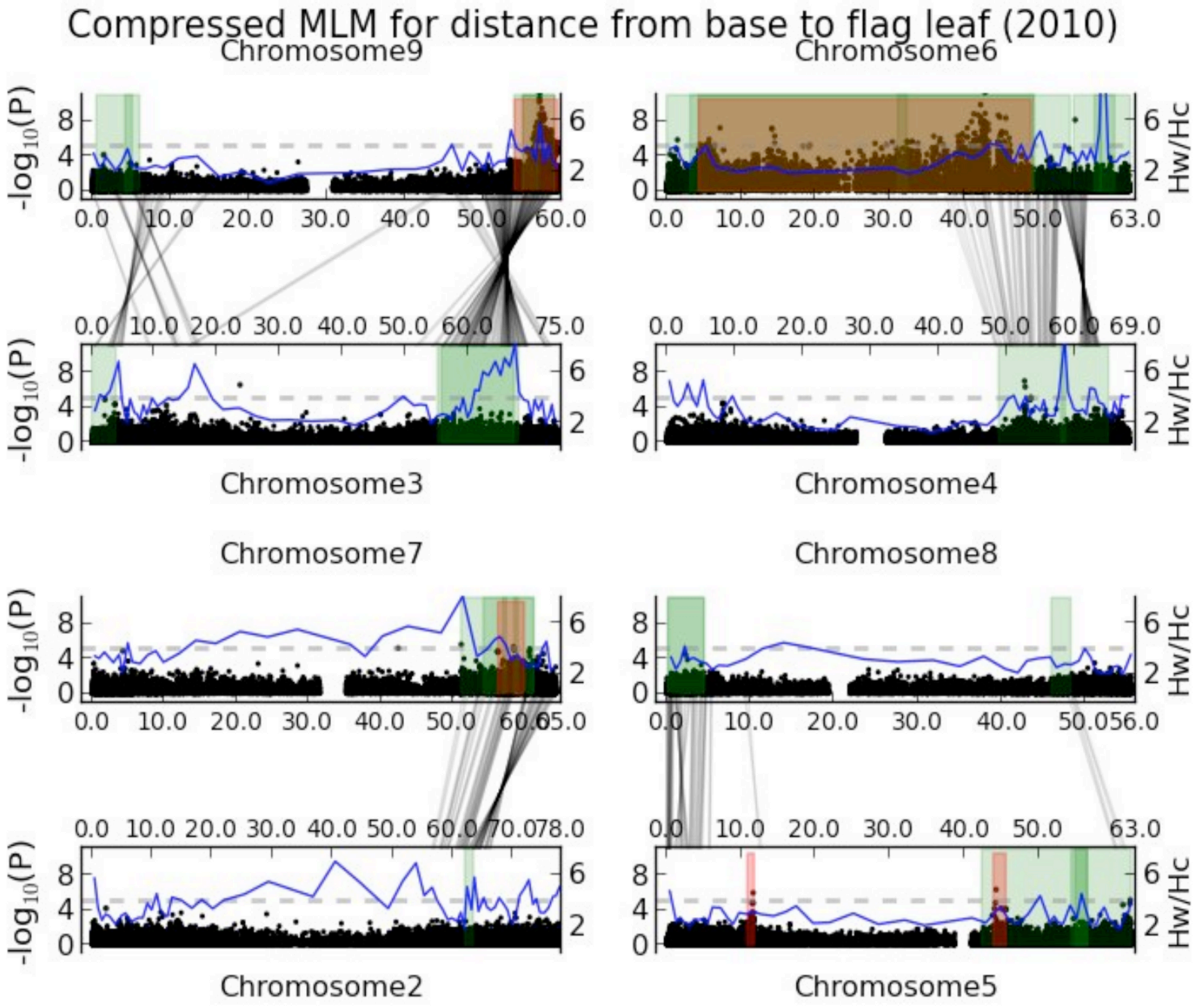

Fig. S17 Genetic correspondence for 2010 base-flag length within sorghum. Chromosome-wide Manhattan plots (Sb02, Sb03, Sb04, Sb05, Sb06, Sb07, Sb08 and Sb09) are plotted with the prior intervals (green area) determined by QTL mapping, the hotspots (red area) identified by GWAS, and the curves of Hw/Hc ratios. Intra-genomic genetic correspondence for QTL intervals are shown on chromosome pairs of Sb03-Sb09, Sb04-Sb06, Sb02-Sb07 and Sb05-Sb08. Gray connecting lines indicate pairs of duplicated genes.

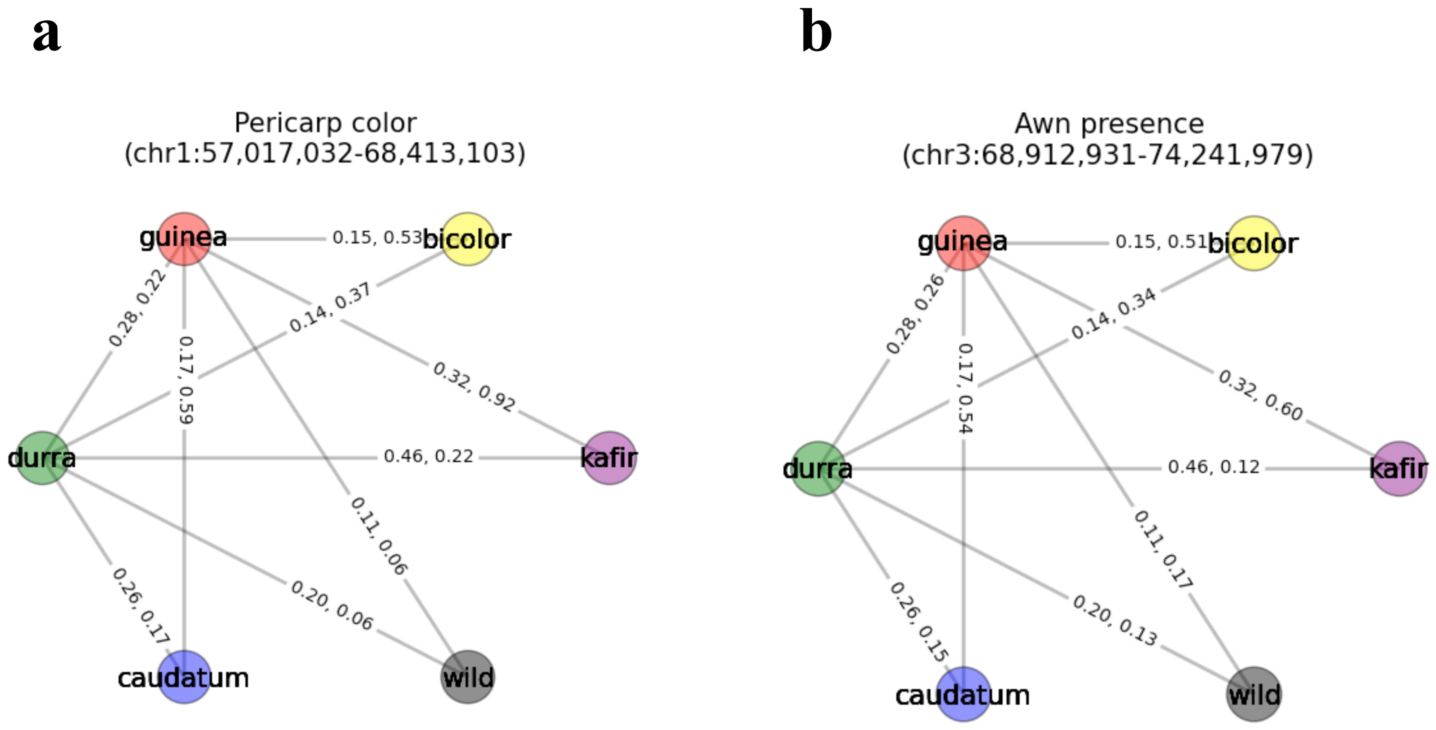

Fig. S18 Genome-wide relationship of 502 worldwide sorghum accessions, including 476 which belong to one of the five main cultivated races and 26 wild types. (a) Population differentiations and frequencies of common two-locus haplotypes for the association peak (S1_61453639) and 7 linked (r^2 >= 0.6) SNPs for pericarp color for pairs of populations. All the connections for guinea and durra are shown. The Fst values paired with frequencies of common two-locus haplotypes are indicated. (b) Population differentiations and frequencies of common two-locus haplotypes for the association peak (S3_72702502) and 18 linked (r^2 >= 0.6) SNPs for awn presence for pairs of populations.
